# A mesoporous metal–organic framework used to sustainably release copper(ii) into reducing aqueous media to promote the CuAAC click reaction†

**DOI:** 10.1039/d2ra04298c

**Published:** 2022-09-22

**Authors:** Pascal Hoffmann, Christian Lherbet, Isabelle Fabing, Marie-Claire Barthélémy, Yann Borjon-Piron, Christophe Laurent, Alain Vigroux

**Affiliations:** LSPCMIB, Université Toulouse 3 Paul Sabatier, UMR CNRS UPS 5068 118 Route de Narbonne 31062 Toulouse France alain.vigroux@univ-tlse3.fr; Institut de Chimie de Toulouse, Université Toulouse 3 Paul Sabatier, ICT-FR CNRS 2599 118 Route de Narbonne 31062 Toulouse France; CIRIMAT, Université Toulouse 3 Paul Sabatier, UMR CNRS UPS INP 5085, 118 Route de Narbonne 31062 Toulouse France

## Abstract

The mesoporous metal–organic framework Cr-MIL-101-NH_2_ (MOF1) has been used to encapsulate, by a simple impregnation method, large amounts of copper sulfate. The resulting loaded material, Cu@MOF1, was successfully employed to slowly release copper(ii) into an appropriate reaction medium in which the reducing agent sodium ascorbate reduces copper(ii) to copper(i), thus allowing the well-known copper(i)-catalyzed alkyne–azide cycloaddition (CuAAC) “click” reaction to proceed in the absence of potentially high local copper(i) concentrations. The use of a MOF-based controlled copper release system such as Cu@MOF1 may be relevant for copper(i)-catalyzed reactions having substrates that could be degraded by potentially high local concentrations of copper(i). The copper chelating ligand TBTA (tris(benzyltriazolylmethyl)amine), a very useful ligand for click chemistry, has been successfully attached to the pores of MOF1. The resulting TBTA-functionalized MOF (MOF3) was compared with its non-functionalized version (MOF1). At copper loadings of *ca.* 3 mmol g^−1^, the results revealed that the performances of the two materials are strikingly similar. Upon immersion in methanol/water (95/5) containing sodium ascorbate, both materials slowly released copper encapsulated in their pores and could be recovered and reused efficiently for up to five reaction cycles without reloading with metal ion, while allowing the CuAAC reaction to proceed with excellent conversion rates and yields.

## Abstract 2

Cr-MIL-101-NH_2_ (MOF1) has been used to encapsulate large amounts of copper sulfate and the resulting loaded material, Cu@MOF1, was successfully used to promote the CuAAC click reaction.

## Graphical abstract

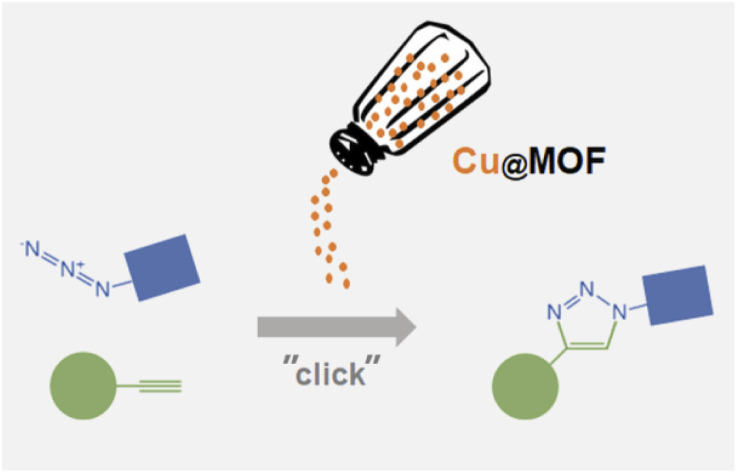

## Introduction

In the last two decades, metal–organic frameworks (MOFs) have attracted considerable interest as a new emerging class of porous materials. Due to their remarkable properties such as high crystallinity and high specific surface areas combined with tunable pore sizes and functionalizable pore walls, MOFs show great potential for a variety of applications, including catalysis, gas storage and separation, chemical sensing, drug delivery, and so on.^1^ In particular, the use of MOFs as a host platform for catalysis has received increasing attention among chemists in recent years.^2^ In this regard, the mesoporous Cr-MIL-101 framework initially published by Férey *et al.*,^3^ appears as a promising ordered porous structure for applications in heterogeneous catalysis. As-synthesized Cr-MIL-101 has coordinated water molecules at its octahedral trinuclear Cr(iii)_3_O building units which can be easily removed in a vacuum and at elevated temperature, thus creating potential Lewis acid sites accessible for reactants. Therefore, due to its open chromium sites, excellent stability, high surface area and large pore volumes, Cr-MIL-101 was tested as heterogeneous catalyst for a wide range of reactions.^4^ In addition to its intrinsic catalytic activity, the MIL-101 framework was also considered as a particularly suitable host matrix for loading catalytically active entities such as molecular metal complexes^5^ and metal nanoparticles.^6^ In this context, the introduction of an amino group on the pore walls of the structure is considered a crucial step to access an ideal starting platform for subsequent post-synthetic functionalizations. The synthesis of the corresponding amine functionalized MOF (Cr-MIL-101-NH_2_) was therefore given special attention.^7^ Indeed, the fact that various organic functional groups, including chelating agents, can be incorporated into the MIL-101 framework *via* post-synthetic modification of Cr-MIL-101-NH_2_ offers the possibility to immobilize active metal centers on a very promising heterogeneous porous support.^8^

Nevertheless, as a Lewis and Brønsted base, the amine function in itself can confer interesting properties to metal–organic frameworks. Amino-functionalized MOFs have often been shown to have noteworthy sorption/desorption capabilities, making them potentially useful materials for various applications. For example, Konar and coworkers reported an amino-functionalized MOF which functions as a syringe pump for the controlled release of iodine as a catalyst^9^ while Falaise *et al.* demonstrated the importance of the presence of amino groups at the pores surface of MOFs for efficient iodine trapping.^10^ Grela & Chmielewski used a simple impregnation strategy to successfully immobilize, by simple adsorption from solution, olefin metathesis catalysts inside an amino-MOF.^5*d*^

**Scheme 1:**
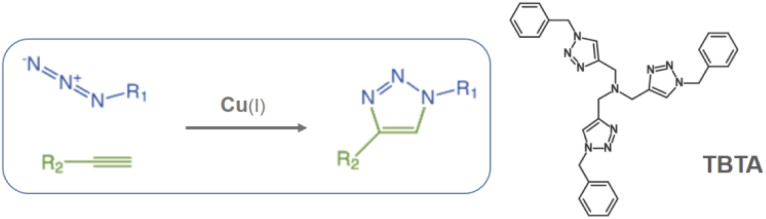
Copper-catalyzed azide–alkyne cycloaddition (CuAAC) and structure of the tris(benzyltriazolylmethyl)amine ligand (TBTA).

In the present work, Cr-MIL-101-NH_2_ was chosen to incorporate a copper(ii) source into its pores using a simple impregnation method. We are interested in developing a simple and inexpensive heterogeneous system that would function as a syringe pump by slowly releasing copper(ii) into a reducing reaction medium allowing the well-known Cu(i)-catalyzed [3 + 2] azide–alkyne cycloaddition (CuAAC) reaction to proceed (Scheme 1).^11^ This reaction has become one of the most attractive examples of the so-called “click chemistry”.^12^ Due to its unique attributes to connect highly functionalized molecules, the CuAAC reaction has attracted considerable attention and found applications in many research fields such as organic synthesis, medicinal chemistry, chemical biology and materials science.^13^

It is well known that copper(i) is the actual catalyst for this transformation.^14^ Accordingly, a large number of organic ligands that are able to stabilize the Cu(i) catalytic center have been developed as homogeneous catalysts in recent years,^15^ the most representative one being the tris-triazole ligand TBTA (tris(benzyltriazolylmethyl)amine, Scheme 1).^15*a*^ Highly nitrogenated ligands such as TBTA and its bulky *tert*-butyl substituted analogues were shown to greatly accelerate the reaction while simultaneously protecting and stabilizing the copper(i) oxidation state.^16^ Despite all these efforts to stabilize the (+I) oxidation state, the use of a Cu(ii) source with addition of a reducing agent in large excess is often preferred. Because this choice allows the reaction to proceed under open-air conditions, synthetic chemists who wish to occasionally implement a CuAAC reaction on a laboratory scale most often select, for practical reasons, a readily available stable copper source such as copper sulfate combined with the reducing agent sodium ascorbate.^11*b*^ However, the copper/ascorbate system is a potent generator of oxygen radicals and other reactive species in air that can have deleterious effects on the substrates and products of the reaction.^17^

In this contribution, we report the preparation and characterization of the CuSO_4_-loaded version of MIL-101-NH_2_ to explore the ability of this material to sustainably release copper(ii) in solvent mixtures in which sodium ascorbate has been solubilized to allow the CuAAC reaction to proceed without damaging potentially sensitive substrates and products.

**Scheme 2:**
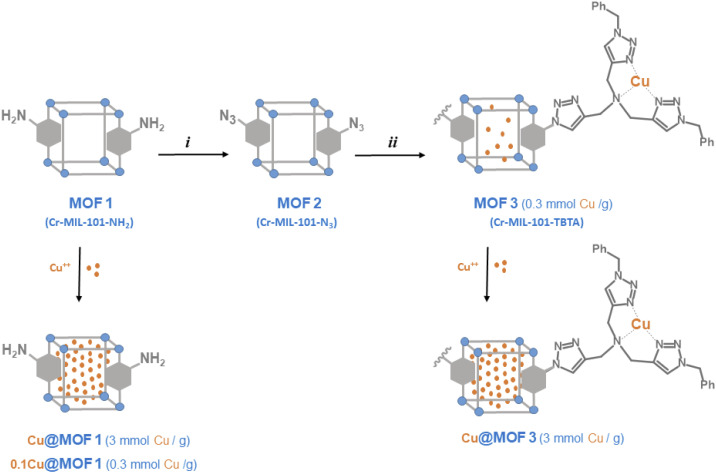
Preparation of MOF materials from Cr-MIL-101-NH_2_ (MOF1), and incorporation of Cu^2+^ ions (i) *t*BuONO, then TMSN_3_, THF; (ii) bis-triazolyl alkyne, Cu(CH_3_CN)_4_PF_6_, THF.

For comparison purposes, we also report the preparation and characterization of the TBTA functionalized MIL-101-NH_2_ to investigate the ability of this new material and its copper loaded version (MOF3 and Cu@MOF3, Scheme 2) to promote the CuAAC reaction. To the best of our knowledge, this work represents the first example of successful incorporation of the TBTA ligand into a metal–organic framework.

## Experimental

### General

All reagents and solvents were purchased from commercial sources (Sigma Aldrich or Alfa Aesar) and were used without further purification. NMR-measurements were performed using a Bruker FT-NMR-spectrometer, Avance 300 (^1^H: 300 MHz; ^13^C: 75 MHz). Analytical UPLC (Ultra Performance Liquid Chromatography) was performed on an UPLC Acquity Waters system, with PDA/MS detectors. FT-IR spectra were recorded on a Thermo Scientific IS50 FT-IR Spectrometer (Thermo Nicolet Corporation) in transmission using KBr pellets.

### Synthesis

Cr-MIL-101-NH_2_ (MOF1) was prepared according a procedure described by Lin and coll.:^18^ chromium(iii) nitrate nonahydrate (99%, 808 mg, 2 mmol), 2-aminoterephthalic acid (99, 365.9 mg, 2 mmol) and sodium hydroxide (200 mg, 5 mmol) were dispersed in deionized water (15 mL) in a 60 mL Schott vial. The solution was stirred for 5 minutes at room temperature, and then heated at 150 °C in an oven for 12 h. After cooling, the green precipitate was collected by centrifugation, washed with DMF (3 × 10 mL) at room temperature, and then with hot ethanol (30 mL) at 100 °C for 24 h in a 60 mL Schott vial. The precipitate was filtered over G3 glass filter, and dried at 80 °C in air.

Cr-MIL-101-N_3_ (MOF2) was prepared from Cr-MIL-101-NH_2_ according a procedure described by Legrand and coll.:^19^MOF1 (300 mg) was placed in a 25 mL round bottom flask with a stir bar and dried at 85 °C (oven) for 14 h. After cooling to room temperature, THF (5 mL) and *tert*-butyl nitrite (*t*BuONO, 2.0 mL) were successively added *via* a syringe. The flask was then cooled to 0 °C (ice bath), and trimethylsilyl azide (TMSN_3_, 1.9 mL) was added dropwise under argon during 15 minutes. The solution was then stirred overnight at room temperature. The green precipitate was collected by centrifugation, washed with THF, filtered over G3 glass filter, and dried at 60 °C for 20 h.

Cr-MIL-101-TBTA (MOF3) was prepared from MOF2 and bis-triazolyl alkyne ligand using CuAAC reaction. Typically, in a 25 mL round bottom flask containing THF (7 mL), Cr-MIL-101-N_3_ (300 mg, 0.356 mmol), alkyne (255 mg, 0.642 mmol) and Cu(CH_3_CN)_4_PF_6_ (80 mg, 2.141 mmol) were stirred for 24 h. Then the resulting green suspension was centrifuged and the resulting green powder was washed with ethyl acetate (3 times) and dried under vacuum.

Yield of post-modification was evaluated by ^1^H-NMR after NaOD/D_2_O hydrolysis of MOF3: a MOF sample (≃10 mg) was suspended in D_2_O (1 mL), and a solution of 40 wt% NaOD in D_2_O (2 μL) was added. The solution was allowed to stand for 16 hours at room temperature, then filtered on alumina (aluminum oxide 90 active neutral from Merck) to remove chromium salts, and the terephthalate derivatives (deprotonated forms) were then analyzed by NMR. For UPLC analysis, the pH was then adjusted to 7.5–8.0 using 6 M hydrochloric acid, and the digestion products were then analyzed by analytical UPLC (UPLC Acquity Waters, PDA/MS detector) with a C18 column (BEH C18, 1.7 μm, 2.1 mm × 100 mm). Water with 0.1% formic acid and methanol with 0.1% formic acid were used as mobile phase A and B, respectively, with a flow rate of 0.5 mL min^−1^, using the following elution method: 0 min, 5% B; 5 min, 100% B; 7 min, 100% B, then returned to initial conditions. The SQD was operated in an electrospray negative ion mode by applying a voltage of 3.5 kV to the ESI capillary and the cone voltage was set at 30 V.

### Incorporation of copper into MOF1 and MOF3: Cu@MOF1 and Cu@MOF3

MOFs 1 and 3 were first activated at 100 °C for 18 h, and the incorporation of the Cu^2+^ ion was performed by adding a solution of copper sulfate (7.5 mg, 0.03 mmol, CuSO_4_·5H_2_O) in deionized water (100 μL) to the activated MOF (10 mg) dispersed in deionized water (100 μL). The solution was allowed to stand at room temperature for 3.5 h, followed by evaporating the water under vacuum. A same procedure using 100 μL of an aqueous CuSO_4_·5H_2_O solution (7.5 mg mL^−1^) has been used to prepare 0.1Cu@MOF1.

### Characterizations

The powder X-ray diffraction (PXRD) patterns were collected with an XPert Pro (Theta–Theta mode) Panalytical powder diffractometer with *λ* (Cu-Kα1, -Kα1) = 1.54059, 1.54439 Å. The specific surface area and pore volume of the solid samples were evaluated from nitrogen adsorption–desorption isotherms at liquid nitrogen temperature (77 K) using a Micromeritics ASAP 2020 apparatus. Specific surface areas were calculated using the BET equation. Pore size distributions were calculated from the desorption branch using the non-local density functional theory (NLDFT) kernel in the MicroActive 3.00 software (carbon, slit pores). Thermogravimetric analysis (TGA) and differential thermal analysis (DTA) were performed simultaneously in flowing synthetic air using a Setaram TG-92 unit. Selected samples were observed by scanning electron microscopy (SEM) using either a JEOL JSM 7800F Prime field-emission microscope operated at 3 kV or a TESCAN VEGA3 microscope operated at 20 kV. In the latter case, an *in situ* Oxford INCA energy-dispersive X-ray spectroscopy (EDS) detector (model X-Max of 50 mm^2^) was used for chemical composition microanalysis.

### General procedure for reaction condition optimization

To a solution of 3-phenyl-1-propyne (0.86 mmol, 1 equiv.) and benzyl azide (1.2 equiv.) in a mixed solvent (1 mL), were added the MOF material (10 mg) containing 0.35 mol% (MOF3) or 3.5 mol% (Cu@MOF1 and Cu@MOF3) copper relative to alkyne (see Table 1) and then sodium ascorbate (10 mg, 6 mol%). The heterogeneous solution was stirred at room temperature for 24 h. The mixture was then transferred to a small centrifuge tube. The precipitate (MOF-catalyst) was washed two times with ethyl acetate. The organic phases were combined and evaporated under reduced pressure. A precise amount of mesitylene was used as internal standard to evaluate yield by ^1^H NMR.

**Table 1:** Optimization of reaction conditions^a^

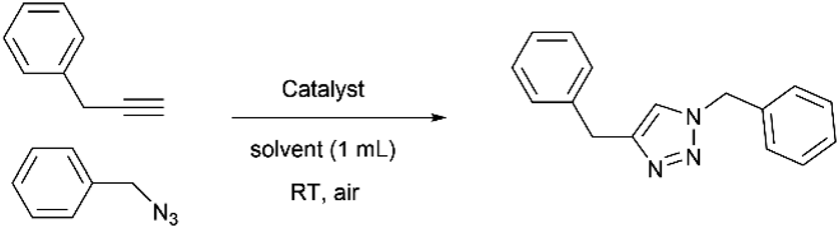				
Entry	Catalyst	Solvent	% conversion^bb^	% estimated yield
1	—	*t*BuOH/H_2_O (70/30)	No reaction	—
2	MOF3^c^	*t*BuOH/H_2_O (70/30)	No reaction	—
3	MOF3	*t*BuOH/H_2_O (70/30)	100 (38)^dd^	92 (17)
4	CuSO_4_·5H_2_O^e^	*t*BuOH/H_2_O (70/30)	75	69
5	MOF3	*t*BuOH/H_2_O (95/5)	35	32
6	MOF3	DMF/H_2_O (95/5)	96 (nd)	91 (11)
7	MOF3	MeOH/H_2_O (95/5)	100 (85)	98 (82)
8	CuSO_4_·5H_2_O^e^	MeOH/H_2_O (95/5)	74	62
9	Cu@MOF1	MeOH/H_2_O (95/5)	100 (100)	99 (96)
10	0.1Cu@MOF1	MeOH/H_2_O (95/5)	78	69
11	Cu@MOF3	MeOH/H_2_O (95/5)	100 (100)	95 (93)
12	Supernatant^f^	MeOH/H_2_O (95/5)	85	73

a Unless otherwise stated, all reactions were performed using 6 mol% sodium ascorbate.

b Conversion estimated by ^1^H NMR based on alkyne (mesitylene was used as internal standard) after reaction runs of 24 h at room temperature.

c No sodium ascorbate added.

d In brackets: “recyclability” (nd: not determined due to partial evaporation of volatile starting materials during DMF removing).

e 0.4 mol% Cu.

f See text.

### Scope of the CuAAC reaction with MOF3 as catalyst

To a solution of alkyne (0.86 mmol, 1 equiv.) and azide (alkyne/azide ratio 1/1.2)‡‡The alkyne/azide ratio was 1/1.05 with the dodecenoyl azide substrate and 1.1/1 with the 1-octyne alkyne substrate. in MeOH/H_2_O (95/5, 1 mL), were added MOF3 (10 mg, 0.35 mol% copper) and then sodium ascorbate (10 mg, 6 mol%). The suspension was stirred at room temperature for 24 h under air. The precipitate (catalyst) was washed two times with ethyl acetate. The organic phases were combined and evaporated under reduced pressure. When necessary, chromatography on a short silica column gave the desired product in high yields.

## Results and discussion

### Preparation and characterization

**Fig. 1:**
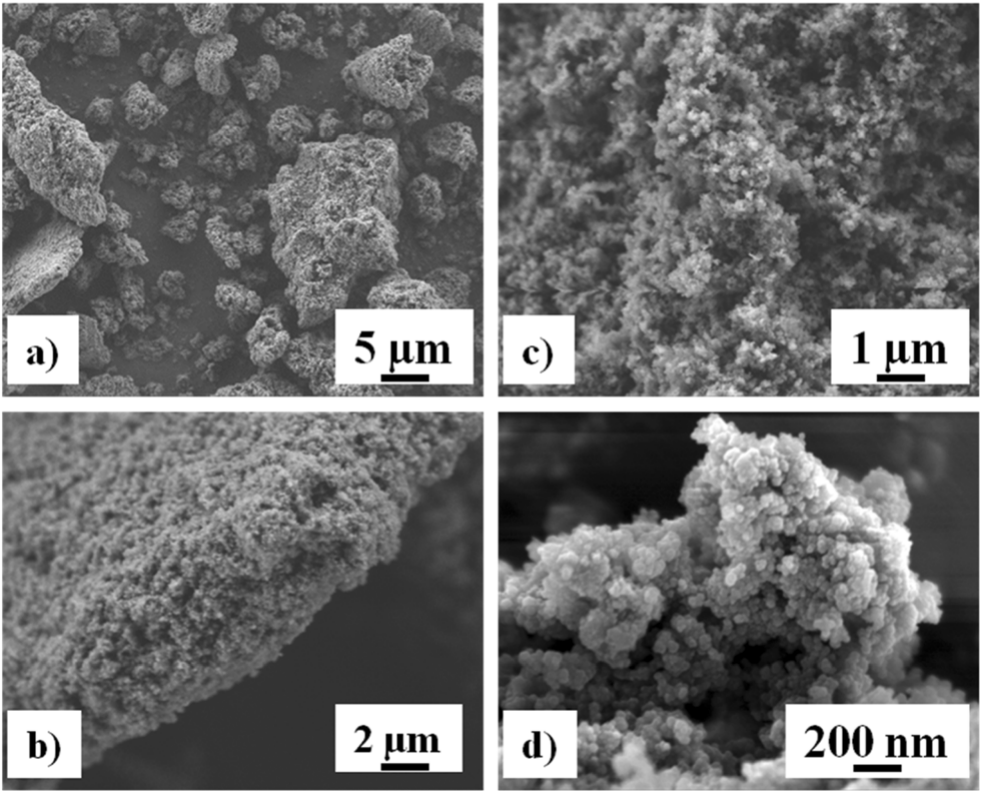
Field-emission SEM images of MOF1 at increasing magnifications (a–d).

The preparation of MOF materials is depicted in Scheme 2. MOF1 (Cr-MIL-101-NH_2_) was prepared from 2-aminoterephthalic acid (H_2_BDC–NH_2_) and chromium nitrate, as already described.^18^ Low magnification field-emission SEM images of MOF1 (Fig. 1a and b) show agglomerates up to about 10 μm in size made up of much finer crystallites. Higher magnification images (Fig. 1c and d) reveal that the crystallites are about 30 nm in size. The crystalline structure of MOF1 was confirmed by PXRD (Fig. S1A†).

The MOF1 sample exhibit a N_2_ sorption isotherm with firstly an important adsorption in the low-pressure domain, thus revealing some microporosity as in type I isotherms, and secondly a narrow hysteresis loop at relative pressures from about 0.5 to 1.0 (Fig. S2A†), which points to a type IV isotherm. Therefore, the isotherm could be described as composite between types I and IV, which are two of the six types of isotherms recognized by the IUPAC classification and characteristic of microporous (pore size below 2 nm) and mesoporous (pore size in the range 2–50 nm) adsorbents, respectively.^20^ The pore size distribution (inset in Fig. S2A†) indeed reveals both micro- and mesoporosity with the larger pores about 3 nm wide. A BET specific surface area of 1620 m^2^ g^−1^ and a total pore volume of 1.22 cm^3^ g^−1^ were calculated.

MOF1 was then treated with *tert*-butyl nitrite (*t*BuONO) and trimethylsilyl azide (TMSN_3_) in THF overnight at room temperature to give the corresponding azide MOF2,^19^ with an acidification yield of approximately 80% as evidenced by ^1^H-NMR after NaOD-digestion that shows the presence of the initial organic ligand H_2_BDC–NH_2_ and the unsubstituted ligand H_2_BDC likely resulting from a deamination side reaction (Fig. S3†). The presence of the azido group of MOF2 was evidenced by FT-IR spectroscopy with a characteristic vibration band at 2123 cm^−1^ corresponding to the asymmetric stretching of N_3_ (Fig. S4†). The 2-azido substituted terephthalate ligand was characterized in its protonated form (H_2_BDC–N_3_) by mass spectrometry in negative mode from the LC-MS chromatogram of the NaOH-digested products of MOF2 (Fig. S5†).

MOF2 was then further post-modified using the copper-catalyzed azide–alkyne cycloaddition (CuAAC) method by reaction with *N*,*N*-bis[(1-benzyltriazol-4-yl)methyl]prop-2-yn-1-amine. This bis-triazolyl alkyne ligand was prepared in three steps, as described previously:^21^ (1) condensation of 3,3-diethoxy-1-propyne and benzyl azide by CuAAC; (2) deprotection of the acetal to afford the aldehyde and (3) reductive amination with propargyl bromide. The PXRD pattern of the TBTA-functionalized MOF (MOF3) is in agreement with the basic three-dimensional architecture of MIL101-family structures,^22^ without significant loss in crystallinity compared to the parent MOF1 (Fig. S1A†).

The ^1^H-NMR spectrum of NaOD-digested MOF3 displays new signals in the aromatic region (7.1–8.1 ppm, Fig. S6†) that can be attributed to triazolic protons at 7.89 and 7.97 ppm, aromatic (terephthalic) protons at 8.02 ppm (dd, *J*_H–H_ = 1.6 and 8.0 Hz), 7.74 ppm (d, *J*_H–H_ = 1.6 Hz) and 7.68 ppm (d, *J*_H–H_ = 8.0 Hz), and phenyl protons at 7.25–7.35 ppm (m). From the ^1^H NMR signal integrations, the ratios of H_2_BDC–NH_2,_ H_2_BDC–N_3_ and H_2_BDC–TBTA were approximately 15, 65 and 20%, respectively. Considering the aperture size of MIL101 windows (12 Å for pentagonal windows and 16 Å for hexagonal windows)^22^ and the size of the TBTA precursor used for the reaction, it is likely that only the azide groups located at the surface of MOF particles reacted, thus leading to a modest post-modification yield of 20%.§§A comparable yield of 15% was obtained for the incorporation of a bulky terpyridine moiety onto the azide tagged Cr-MIL-101-N_3_ using the same “click’’ post-functionalization as that described in the present work. See ref. 8*b*. This low grafting yield of the TBTA group is confirmed by the FT-IR spectrum of MOF3 which shows that the characteristic band of the azido group at 2122 cm^−1^ is still present, but with a somewhat reduced intensity compared to MOF2 (Fig. S4†). The UPLC/MS in negative mode of NaOH-digested MOF3, displaying two major peaks in an 82/18 ratio with values of *m*/*z* 206.0 and 604.3 corresponding to HBDC^−^-N_3_ and HBDC^−^-TBTA, respectively, confirmed that MOF2 was partially modified successfully by TBTA in the click reaction (Fig. S7†). As expected, the bulky TBTA group of MOF3 leads to a marked decrease in its total pore volume (from 1.22 to 0.79 cm^3^ g^−1^) and BET specific surface area (from 1620 to 730 m^2^ g^−1^) as compared to MOF1 (Fig. S2B†). The observation of the low-pressure domain and the pore size distribution (inset in Fig. S2B†) reveals a lower microporosity than for MOF1, without any really significant change for the mesopore domain. These data thus show that the TBTA-functionalized MOF remains a significantly porous material.¶¶Considering the density of copper sulfate pentahydrate (2.286 g cm^−3^)|| and the total pore volume of MOF1 and MOF3 (1.22 and 0.79 cm^3^ g^−1^, respectively), the maximum copper storage capacity can be estimated at 28 mg of CuSO_4_·5H_2_O for 10 mg of MOF1 and 18.2 mg of CuSO_4_·5H_2_O for 10 mg of MOF3. Cu@MOF1 and Cu@MOF3 were prepared with a copper sulfate loading level of 7.5 mg per 10 mg of MOF (see Experimental section), which represents only 26.8 and 41.2% of the total loading capacity of MOF1 and MOF3, respectively. Inductively coupled plasma (ICP) analysis indicated that a fraction of copper originating from the CuAAC reaction used to prepare MOF2 was still contained in MOF3 at about 0.3 mmol g^−1^ with a Cr/Cu atomic ratio of 4.85||CRC Handbook of Chemistry and Physics, ed. Haynes M. William, CRC Press, Boca Raton, FL, 92 edn, 2011..

Encapsulation of Cu^2+^ ions into activated MOFs was performed by simply suspending MOF1 or MOF3 in a solution of copper(ii) sulfate in water at room temperature for 3.5 h, followed by water removing. The corresponding loaded materials, Cu@MOF1 and Cu@MOF3, were anticipated to feature a copper content of *ca.* 3 mmol g^−1^ (*i.e.* 0.03 mmol per 10 mg of MOF), which corresponds to a copper concentration ten times higher than that measured for MOF3 (0.3 mmol g^−1^). For comparison purposes, MOF1 was also loaded so that it contains approximately the amount of copper found in MOF3. The corresponding material is referred to as 0.1Cu@MOF1.

Analysis of the PXRD patterns indicates that the crystalline structure of the MOF is still maintained after the encapsulation process (Fig. S1B†). The PXRD pattern of Cu@MOF1 contains no obvious (or only very weak) peaks characteristic of crystalline CuSO_4_·5H_2_O, indicating that copper sulfate is well dispersed in the internal pores of the MOF host instead of residing on the external surface. By contrast, the PXRD pattern for a hand-milled mixture of MOF1 and CuSO_4_·5H_2_O (referred to as MOF1/CuSO_4_) containing the same proportion of copper as Cu@MOF1 clearly reveals the corresponding sub-patterns (Fig. S1B†).

**Fig. 2:**
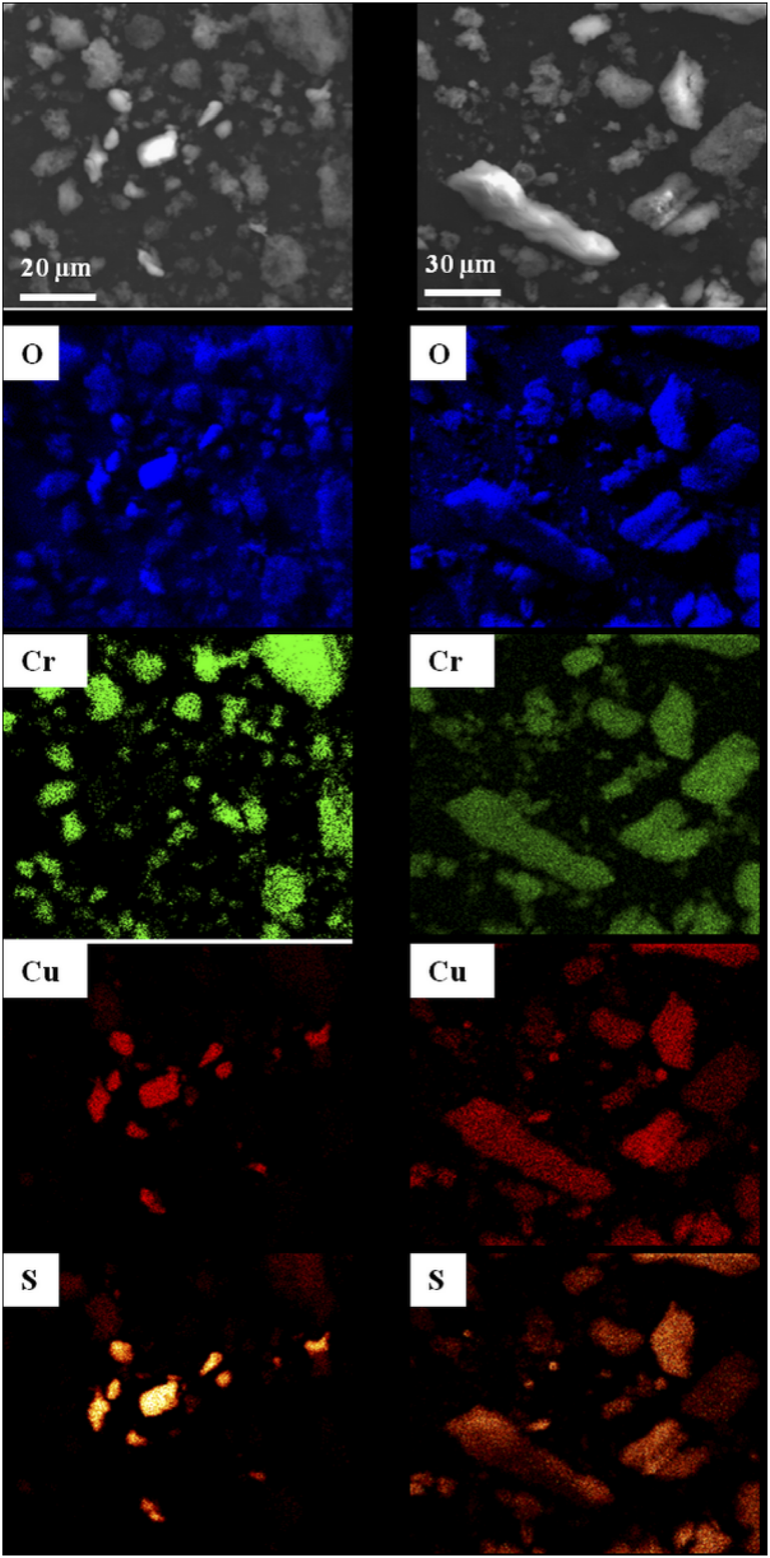
SEM images and the corresponding EDS elemental maps (oxygen, chromium, copper and sulfur) for a mixture of MOF1 and CuSO_4_·5H_2_O (left panel) and Cu@MOF1 (right panel).

SEM observations of MOF1/CuSO_4_ and Cu@MOF1 were combined with EDS analyses mapping for the elements oxygen, chromium, copper and sulfur (Fig. 2), in order to determine the distribution of the copper sulfate throughout the sample with respect to MOF1. For MOF1/CuSO_4_ (left panel in Fig. 2), the copper sulfate particles are clearly distinct from the MOF particles. By contrast, for Cu@MOF1 (right panel in Fig. 2), all four elements are uniformly detected in all investigated particles, which could reveal a uniform distribution of copper sulfate on and probably within the MOF particles, which would be in line with the PXRD results.

**Fig. 3:**
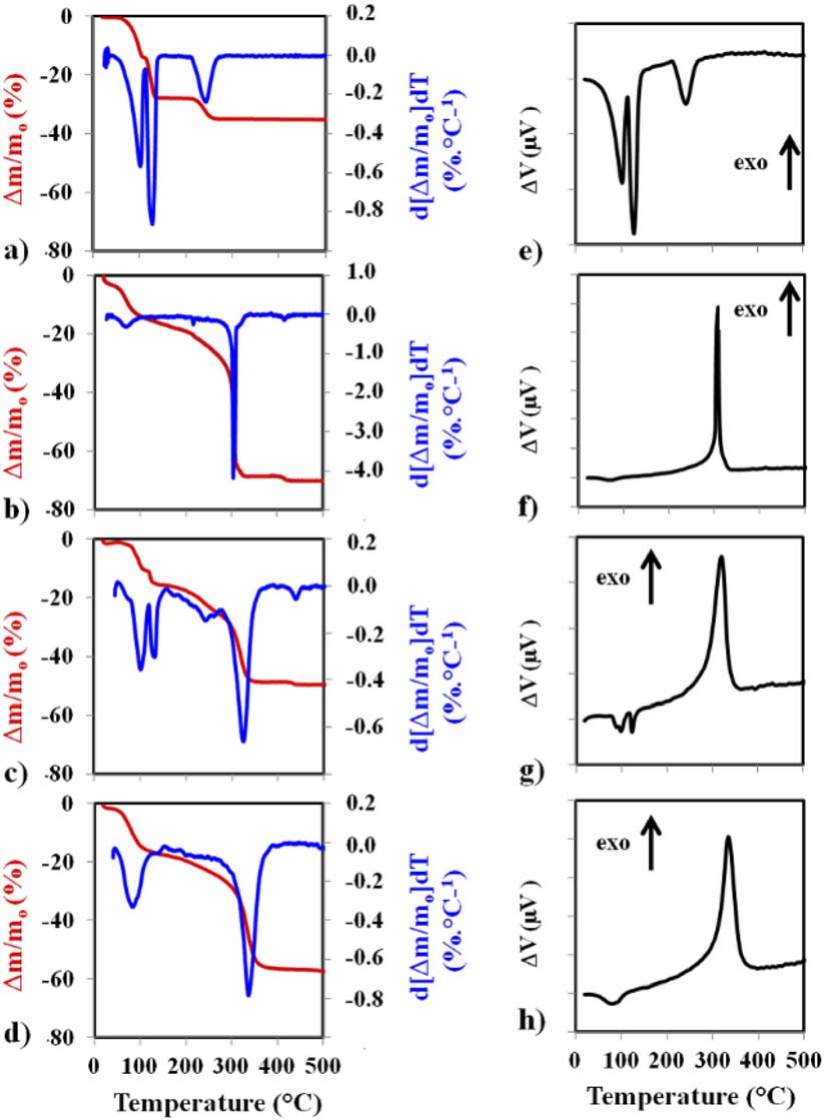
The TGA curves (red) and the calculated DTG curves (blue) for CuSO_4_·5H_2_O (a), MOF1 (b), MOF1/CuSO_4_, the mixture of CuSO_4_·5H_2_O and MOF1 (c) and Cu@MOF1 (d). The corresponding DTA curves are on the right panel (e–h).

Thermal analysis curves recorded in flowing synthetic air are shown in Fig. 3. On the left panel of Fig. 3b–d are the TGA curve (red) and the calculated differential thermogravimetry (DTG) curve (blue). The corresponding DTA curves are on the right panel of Fig. 3e–h. The TGA and DTG curves for CuSO_4_·5H_2_O (Fig. 3a) show the progressive dehydration, in three steps (−2H_2_O, −2H_2_O, –H_2_O), leading to CuSO_4_, which is known to be thermally stable up to over 650 °C. The corresponding DTA peaks (Fig. 3e) are all endothermic. For MOF1 (Fig. 3b), after some desorption occurring below 100 °C, the decomposition occurs in several steps, the most intense one by far, also very sharp, at about 303 °C.

The corresponding DTA peak (Fig. 3f) is exothermic. For MOF1/CuSO_4_, the TGA and DTG curves (Fig. 3c) closely resemble a superimposition of the respective patterns. In particular, the first two dehydration events of CuSO_4_·5H_2_O and the sharp decomposition peak of MOF1 are clearly detected. There may be some interference between the third dehydration and the MOF decomposition, which could account for the slight shift of the latter peak (325 °C *vs.* 303 °C). Accordingly, the corresponding DTA curve (Fig. 3g) shows two endothermic peaks and one exothermic peak, the latter being wider than that observed for MOF1. For Cu@MOF1, the TGA and DTG curves (Fig. 3d) do not show the pattern for free CuSO_4_·5H_2_O (notably, the third dehydration peak is absent), although some residual presence cannot be ruled out. The MOF decomposition peak is different from that for the free MOF1 and has shifted slightly to higher temperatures (337 °C). The DTA curve (Fig. 3h) shows something similar. Although a precise determination of the sequence of events would require further studies, it can be inferred from the present results that Cu@MOF1 does not thermally behave like a simple mixture and that some CuSO_4_·5H_2_O has indeed been encapsulated inside the pores.

### Catalytic/delivery studies

Cu@MOF1 and MOF3 were first assayed for their ability to promote a generic CuAAC reaction, *i.e.* cyclization of benzyl azide and 3-phenyl-1-propyne (Table 1) using different water-mixed solvents at room temperature for 24 h. Not surprisingly, with both materials no triazole formation was observed without the use of a copper(ii) reductant (Table 1, entry 2), attesting the absence of an effective concentration of the catalytic reactive species Cu^+^ in the reaction medium. Among the different water-mixed solvents used in the presence of the reducing agent ascorbate, it appeared that protic solvent methanol containing 5% water generally led to higher yields and conversions than DMF or *tert*-butanol with the same water content. Under these conditions, MOF3, Cu@MOF1 and Cu@MOF3 were all found to be effective in promoting the cycloaddition reaction, all three materials leading to a total conversion of the limiting alkyne reagent with estimated triazole yields of 98, 99 and 95%, respectively (Table 1, entries 7, 9 and 11).

It is reasonable to assume that the observed conversions result from the ability of the solvent to desorb copper sulfate from the pore surface of MOFs. Indeed, copper sulfate is held inside the MOFs by reversible non-covalent interactions so that desorption of the latter is an obvious possibility, especially with the solvent mixtures usually used to solubilize the reducing agent sodium ascorbate (most often polar solvents mixed with water). An attempt was therefore made to determine if the catalytic reaction observed with Cu@MOF1 takes place primarily in the bulk solvent mixture (homogeneous reaction), as a result of copper release, or if it occurs inside the pores of the catalyst instead (heterogeneous reaction). To this end, a preliminary run was conducted using freshly loaded Cu@MOF1 as catalyst and benzyl azide and 3-phenyl-1-propyne as reagents. Triazole product was obtained from this initial run with 100% conversion and 99% yield (Table 1 entry 9). The solid catalyst was recovered by centrifugation, washed twice with ethyl acetate and then soaked at room temperature in a freshly prepared methanol/water (95/5) mixture containing sodium ascorbate but not the azide and alkyne reagents. After 24 hours soaking, the solid material was removed by centrifugation, and washed with ethyl acetate (to be used in a next run) while the supernatant soaking solution was recovered to be tested in the absence of the solid catalyst. As shown in Table 1 (entry 12), a significant triazole formation occurred (with 85% conversion and 73% yield) from the supernatant solution when subjected to the same reagents and reaction conditions while the recovered solid catalyst exhibited no significant decrease in activity in its second run (100% conversion and 96% yield). These experiments clearly show that the conversions observed with the loaded material Cu@MOF1 result primary from a sustained release of copper in the methanol–water mixture followed by homogeneous reaction.

The Cu(ii)-Ascorbate association, which is essential to catalyze the CuAAC reaction, most likely requires that copper sulfate desorbs from the solid support to the aqueous ascorbate solution. Desorption, and thus catalysis, should therefore increase with the water content of the solvent used. This is what is observed for the performance of MOF3 in *tert*-butanol. In *tert*-butanol containing 30% water, the conversion is 100% (Table 1, entry 3) while it is only 35% when the water content drops to 5% (Table 1, entry 5). This difference probably reflects the superior ability of water to desorb copper sulfate compared to *tert*-butanol. Also, as opposed to *tert*-butanol, methanol certainly plays an active role with water in desorbing copper sulfate. Indeed, higher conversions are obtained (100% *vs.* 35%) when *tert*-butanol is replaced by methanol in the presence of 5% water (Table 1, entries 5 and 7).

Although the observed conversions are essentially the result of the desorption of copper, the presence of a heterogeneous support such as MOF3 appears to be advantageous for the CuAAC reaction. MOF3 containing 0.35 mol% copper exhibits higher conversions in *t*-BuOH/H_2_O 70/30 than the corresponding homogeneous reaction performed with 0.4 mol% copper (Table 1, entries 3 and 4). A better performance of MOF3 relative to the homogeneous reaction is also observed in MeOH/H_2_O 95/5 (Table 1, entries 7 and 8). On the other hand, there is no significant difference in conversion between the homogeneous reactions when the latter are performed in the two solvent mixtures (Table 1, entries 4 and 8).

**Fig. 4:**
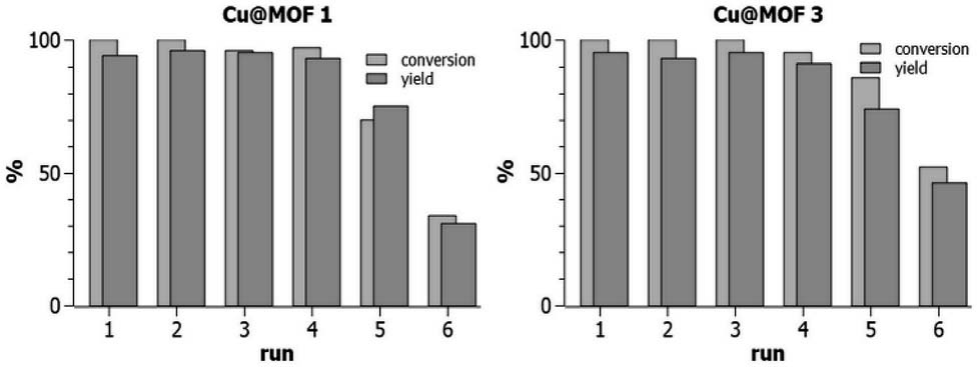
“Recyclability” (see text) of Cu@MOF1 and Cu@MOF3 in the CuAAC reaction between benzyl azide and 3-phenyl-1-propyne at room temperature in methanol/water 95/5 (after each run of 24 h, the solid material was centrifuged, washed with ethyl acetate, dried under vacuum, and then used directly in the next run). Runs were done in duplicate with error < 2% for runs 1–4 and error < 5% for runs 5 and 6.

In the following, we use the term “recyclability” to express the ability of MOF3, Cu@MOF3 and Cu@MOF1 to release Cu(ii) in a sustainable manner. The release rate of Cu(ii) into the reaction medium containing the ascorbate reductant depends on the nature and composition of the solvent mixture used. A significant copper release occurred during the first reaction cycle of MOF3 in the 70/30 *t*-BuOH/H_2_O mixture (24 h at room temperature) as evidenced by the low conversion rate obtained at the second cycle (38%, Table 1, entry 3). This sharp decrease can be attributed to the ability of water to remove, in a single cycle, most of the copper sulfate contained in MOF3. Solvent mixtures in which copper sulfate is most soluble are likely to promote substantial desorption of copper from the very first cycle, thus radically altering the ability of the material to sustainably release copper in subsequent cycles. Since the best “recyclability” of MOF3 is obtained in the 95/5 MeOH/H_2_O solvent mixture, which also gives better yields and conversion rates (Table 1, entry 7), we investigated the “recyclability” of Cu@MOF1 and Cu@MOF3 (*i.e.*, their ability to be used as a copper source during several catalytic cycles) in this solvent mixture using the same model reaction (Table 1). After each run of 24 h, the loaded materials were centrifuged, washed with ethyl acetate, dried under vacuum, and then used directly in the next run. The “recycling experiments” presented in Fig. 4 clearly show that Cu@MOF3 and Cu@MOF1 exhibit a closely similar “recyclability” profile during the first five cycles. Within the first four catalytic runs, conversions of 95–100% were obtained with yields of 91–96% for both materials. After four runs, the two loaded materials still performed similarly with identical yields (74–75%) but with slightly different conversions (70% for Cu@MOF1 and 86% for Cu@MOF3). A significant decrease in conversion and yield is observed at the sixth run for both MOFs.

It is important to note that MOF3 and its copper-loaded version, Cu@MOF3, contain about 65% of azido groups that did not react with the bulky TBTA precursor (the bis-triazolyl alkyne ligand) during the preparation of MOF3. Thus, from the first catalytic runs of MOF3 and Cu@MOF3, one may expect a significant CuAAC click reaction occurring between the 3-phenyl-1-propyne substrate (which is sterically much less bulky than the bis-triazolyl alkyne ligand) and the unreacted azide groups present in both MOFs. The FT-IR spectrum of MOF3 recorded after a first catalytic run clearly shows that the intensity of the residual azido band at 2122 cm^−1^ further decreased after this run (Fig. S4†). This concomitant post-synthetic reaction is anticipated to produce benzylated triazole units covalently bound inside the pores of MOF3 and Cu@MOF3. It is therefore questionable whether those triazole units possibly attached at the pores surface contribute significantly to the performance of the corresponding MOFs. The closely similar “recyclability” profiles of Cu@MOF3 and Cu@MOF1 (Fig. 4) suggest that the triazole units, if present, do not actually play a significant role, at least for copper loadings as high as 3 mmol g^−1^.

Overall, these results suggest (i) that, in the loading conditions used, Cu@MOF3 and Cu@MOF1 contain similar amounts of encapsulated copper¶ and (ii) that encapsulated copper is sustainably delivered from the pores of both materials upon immersion in methanol/water (95/5) at similar desorption rates. The “recycling” results also suggest that the covalent fixation (*ca.* 20%) of the copper chelating agent TBTA on the MIL-101-NH_2_ framework does not lead to a significantly better “recycling” performance than that observed with the unmodified MIL-101-NH_2_ framework. This is likely due to the high copper loading (*ca.* 3 mmol g^−1^) resulting from the pore volume capacity of MOF1 and MOF3, which probably masks the positive catalytic effect usually observed with the TBTA ligand.

**Scheme 3:**
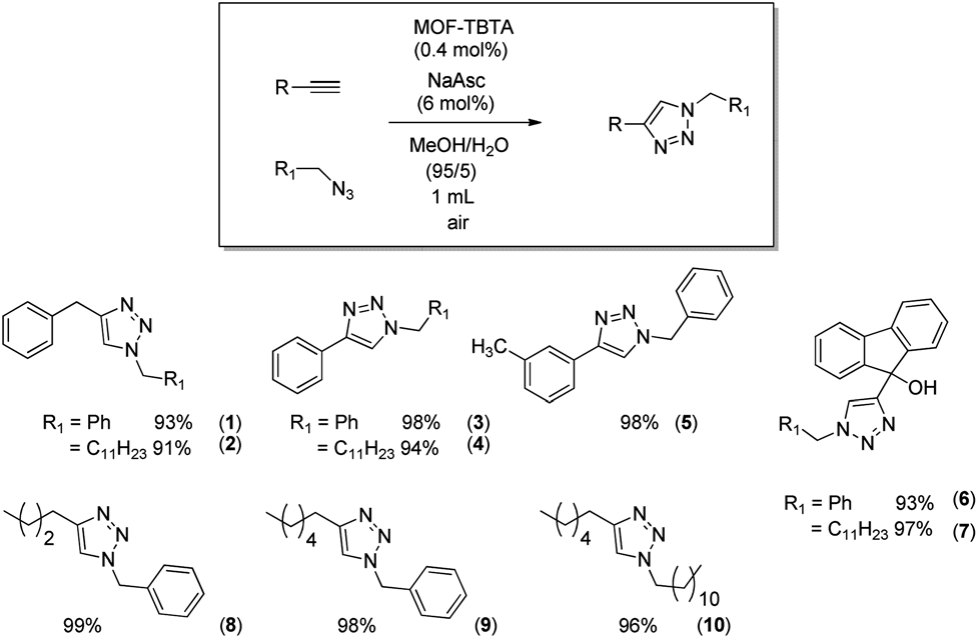
Scope of the reaction with MOF3 (0.4 mol% Cu) as catalyst.

The anticipated positive effect of TBTA is expected to be more pronounced at lower catalyst loadings. Indeed, at copper loadings ten times lower (*ca.* 0.3 mmol g^−1^), the “recycling” performance of the TBTA-conjugated MOF (MOF3, Table 1, entry 7) is markedly better than that of MOF1 loaded with a similar amount of copper (0.1Cu@MOF1, Table 1, entry 10). This prompted us to use MOF3 in MeOH/H_2_O (95/5) to evaluate the scope of the CuAAC reaction by extending it to a variety of synthesized or commercially available azide and alkyne reagents. The aromatic and/or aliphatic nature of the substrates studied did not affect the reaction. In all cases, the expected products were obtained with high yields ranging from 93% to 99% (Scheme 3).

## Conclusions

The spacious pores of MOF1 (Cr-MIL-101-NH_2_), in the range 0.7–3 nm, and the sorption/desorption properties of its amino-functionalized framework have been successfully used to encapsulate, by a simple impregnation method, large amounts of copper sulfate. The resulting loaded material, Cu@MOF1, was found to sustainably release copper in solvent mixtures in which the reducing agent sodium ascorbate is soluble (typically, polar solvents mixed with some water). In appropriate aqueous media, Cu@MOF1 therefore has properties that allow it to be used as a convenient heterogeneous copper(ii) source to promote, in the presence of sodium ascorbate, copper(i)-catalyzed reactions such as the CuAAC reaction. At a copper sulfate loading level of 7.5 mg per 10 mg of material, which represents only about 27% of the total loading capacity of MOF1, Cu@MOF1 could be recovered and reused efficiently for up to five reaction cycles without reloading with metal ion, while allowing the CuAAC reaction to proceed with excellent conversion rates and yields. The copper chelating ligand TBTA has been covalently incorporated into the pores of MOF1 by using a click post-synthetic modification of the azide-functionalized version of MOF1. It appears that the so-obtained material containing *ca.* 20% TBTA (MOF3) does not exhibit a significantly improved performance relative to the corresponding loaded version of MOF1. At copper loadings as high as 3 mmol g^−1^, the catalytic/recycling results obtained with Cu@MOF1 are indeed comparable to those obtained with the much more sophisticated and hard-to-obtain material Cu@MOF3. The beneficial effect of the TBTA ligand is noticeable only at an initial copper loading ten times lower (*i.e.* 0.3 mmol g^−1^). We believe that the controlled copper(ii) release system used in the present study may be particularly relevant for reactions having sensitive substrates that could be damaged by the high local copper(i) concentrations that may occur when the catalyst is introduced manually into the reaction medium, regardless of whether this introduction is slow or not.

## Conflicts of interest

There are no conflicts to declare.

## Supplementary Material

RA-012-D2RA04298C-s001
