# Supplementary material for: A mesoporous metal–organic framework used to sustainably release copper(ii) into reducing aqueous media to promote the CuAAC click reaction
Source: RSC Adv. 2022 Sep 22;12(41):26825–33. doi: 10.1039/d2ra04298c (PMC9494208; doi:10.1039/d2ra04298c)
Supplement: RA-012-D2RA04298C-s001 [file RA-012-D2RA04298C-s001.pdf]

Electronic Supplementary Material (ESI) for RSC Advances.
This journal is © The Royal Society of Chemistry 2022

SUPPLEMENTARY INFORMATION

A mesoporous metal-organic framework used to sustainably release copper(II) into reducing aqueous media to promote the CuAAC click reaction

Pascal Hoffmann,[a] Christian Lherbet,[a] Isabelle Fabing,[b] Marie-Claire Barthélémy,[c] Yann Borjon-Piron,[c] Christophe Laurent[c] and Alain Vigroux*,[a]

a. LSPCMIB, Université Toulouse 3 Paul Sabatier, UMR CNRS UPS 5068, 118 Route de Narbonne, 31062 Toulouse, France.

b. Institut de Chimie de Toulouse, Université Toulouse 3 Paul Sabatier, ICT-FR CNRS 2599, 118 Route de Narbonne, 31062 Toulouse, France.

c. CIRIMAT, Université Toulouse 3 Paul Sabatier, UMR CNRS UPS INP 5085, 118 Route de Narbonne, 31062 Toulouse, France.

✉ Alain Vigroux
alain.vigroux@univ-tlse3.fr

**SI-2**	**Fig. S1** – Simulated and experimental PXRD patterns
**SI-3**	**Fig. S2** – Adsorption-desorption isotherms of **MOF1** and **MOF3** and the corresponding pore size distributions
**SI-4**	**Fig. S3** – ^{1}H-NMR spectrum of NaOD-digested **MOF2**
**SI-5**	**Fig. S4** – FT-IR spectrum of **MOF2** and **MOF3**
**SI-6**	**Fig. S5** – UPLC chromatogram with the MS spectra of NaOH-digested **MOF2**
**SI-7**	**Fig. S6** – ^{1}H-NMR spectrum of NaOD-digested **MOF3**
**SI-8**	**Fig. S7** – UPLC chromatogram with the MS spectra of NaOH-digested **MOF3**
**SI-9**	**Table S1.** "Recyclability" of **Cu@MOF1** and **Cu@MOF3** materials
**SI-10 – SI-11**	^{1}H- and ^{13}C-NMR data of compounds **4 – 7, 8 – 10**
**SI-12 – SI-17**	^{1}H- and ^{13}C-NMR spectra of compounds **4 – 7, 8 – 10**

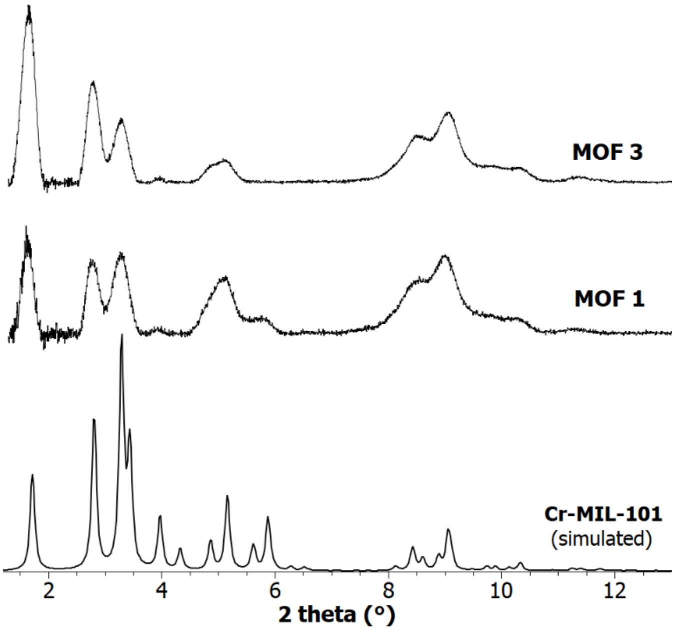

A

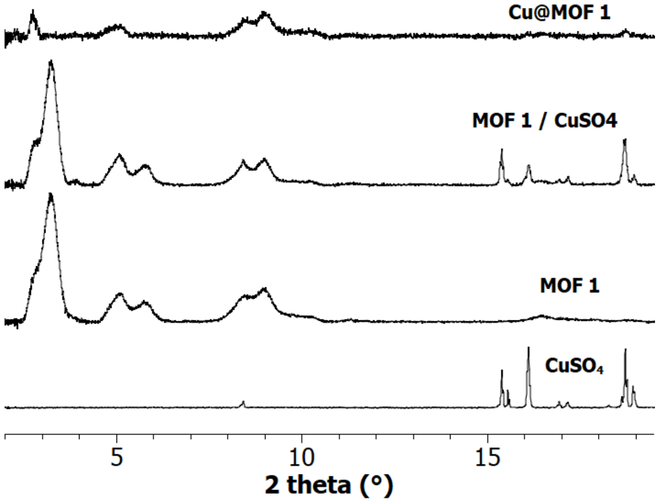

B

Figure S1. A. PXRD patterns simulated (1.54056 Å) from the crystal data of **Cr-MIL-101**, and recorded for a sample of **MOF 1** (Cr-MIL-101-NH_2) and **MOF 3** (Cr-MIL-101-TBTA). **B.** PXRD patterns recorded for a sample of $CuSO_4.5H_2O$, **MOF1**, **MOF1/$CuSO_4$** and **Cu@MOF1**.

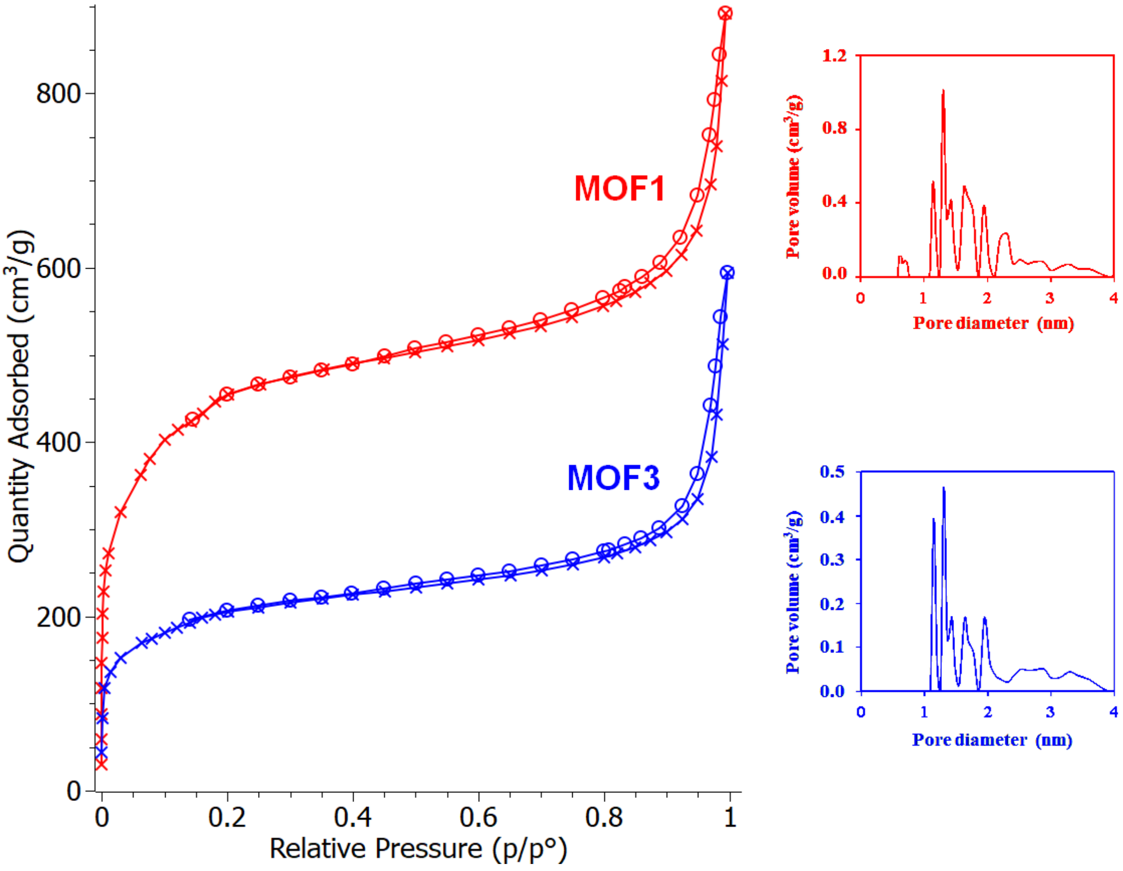

Figure S2. N_2 adsorption-desorption experiments and calculated pore size distribution of Cr-MIL-101-NH_2 (**MOF1**, red) and **MOF3** (blue). N_2 adsorption desorption isotherms were performed on a Micromeritics ASAP 2020 apparatus and measured at liquid N_2 temperature (77 K).

A known amount of sample (about 50 mg) was loaded into the sample cell and degassed under vacuum (10^{-5} Torr) at 150 °C for 11 h. The BET specific surface areas (1620 m^2/g for **MOF1** calculated in the P/P° range 0.0617-0.2016 and 730 m^2/g for **MOF3** calculated in the P/P° range 0.0641-0.2015) and pore size distribution were calculated from the desorption isotherms.

- Calculated total pore volume (**MOF1** : 1.22 cm^3/g, **MOF3** : 0.79 cm^3/g).
- Calculated micropore volume and micropore surface area (**MOF1**: 0.560 cm^3/g and 737 m^2/g, respectively, **MOF3**: 0.252 cm^3/g and 262 m^2/g, respectively).

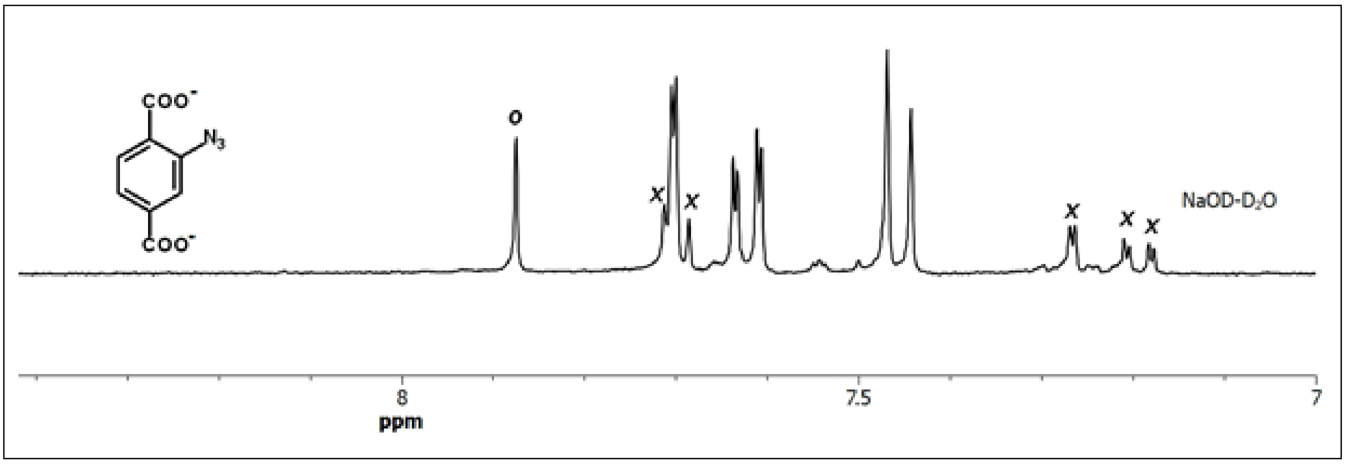

Figure S3. 7.0-8.4 ppm-range ^{1}H-NMR spectrum (300 MHz, NaOD/D_2O or d^6-DMSO) of the digested **MOF 2**. For ^{1}H-NMR measurements, a MOF sample (≃ 10 mg) was suspended in D_2O (1 mL), and a solution of 40 wt% NaOD in D_2O (2 µL) was added. The solution was allowed to stand for 16 hours at room temperature, filtered on alumina (aluminum oxide 90 active neutral from Merck) to remove chromium salts, and then analyzed by NMR. It should be noted the presence of ≃ 20 % of remaining BDC-NH_2 (signals denoted "x"), and of BDC (signal denoted "o") probably due to a deamination reaction.

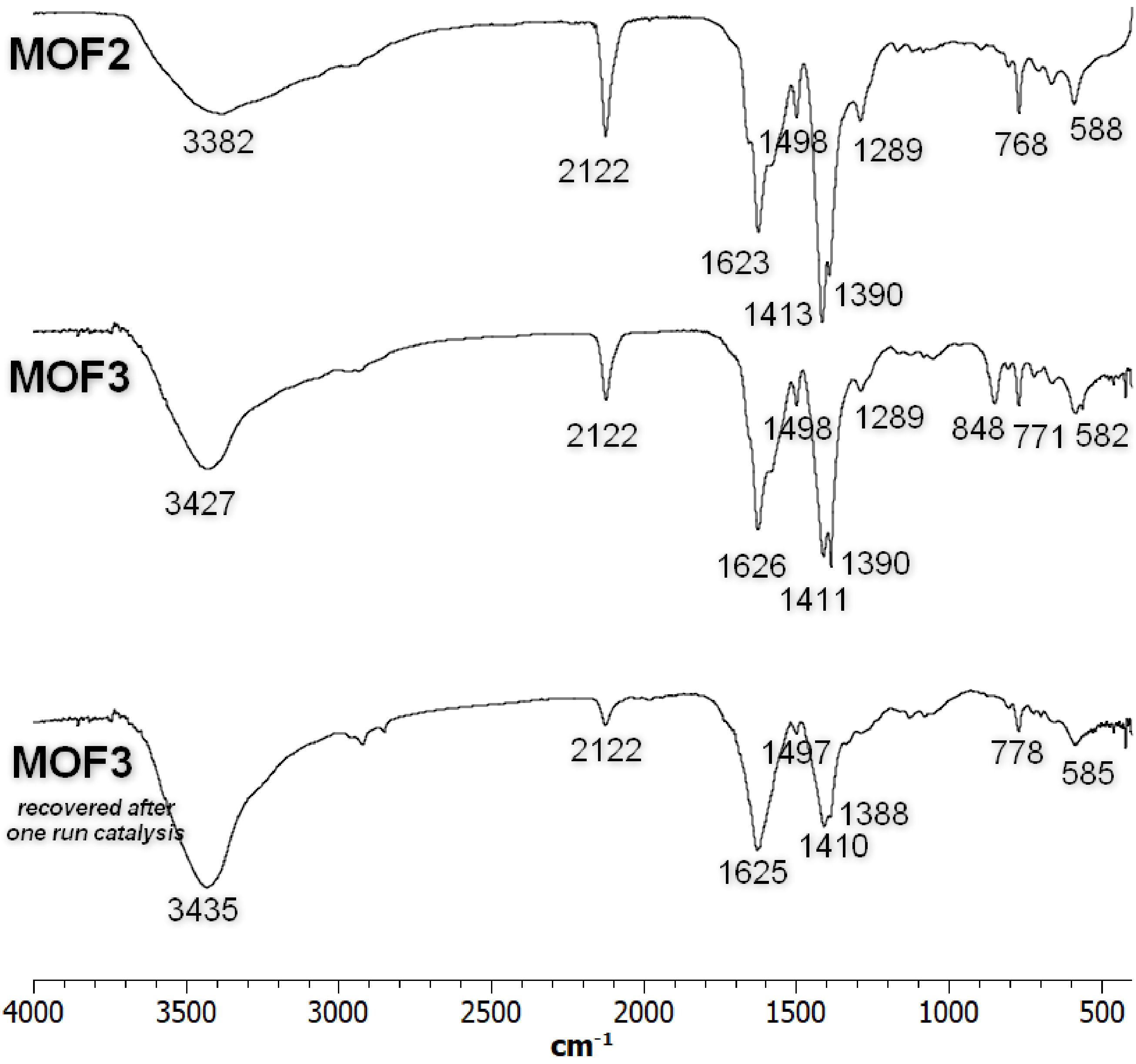

Figure S4. FT-IR spectra of **MOF 2** and **MOF 3** (as synthesized and after one reaction run) displaying azido bands at 2122 cm^{-1}.

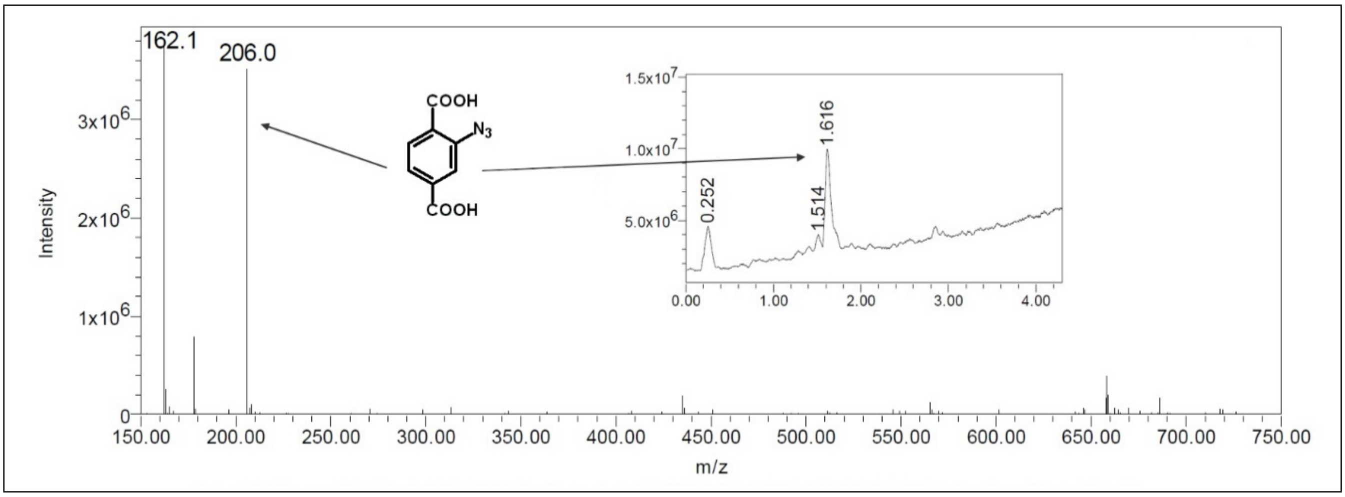

Figure S5. UPLC chromatogram (inset plot) of the digested products in approx. 0.02 M NaOH of **MOF 2** (retention time: 1.616 min) with the mass spectrum acquired in ESI negative mode corresponding to 2-azidoterephthalic acid ($C_8H_5N_3O_4$, MW=207.143).

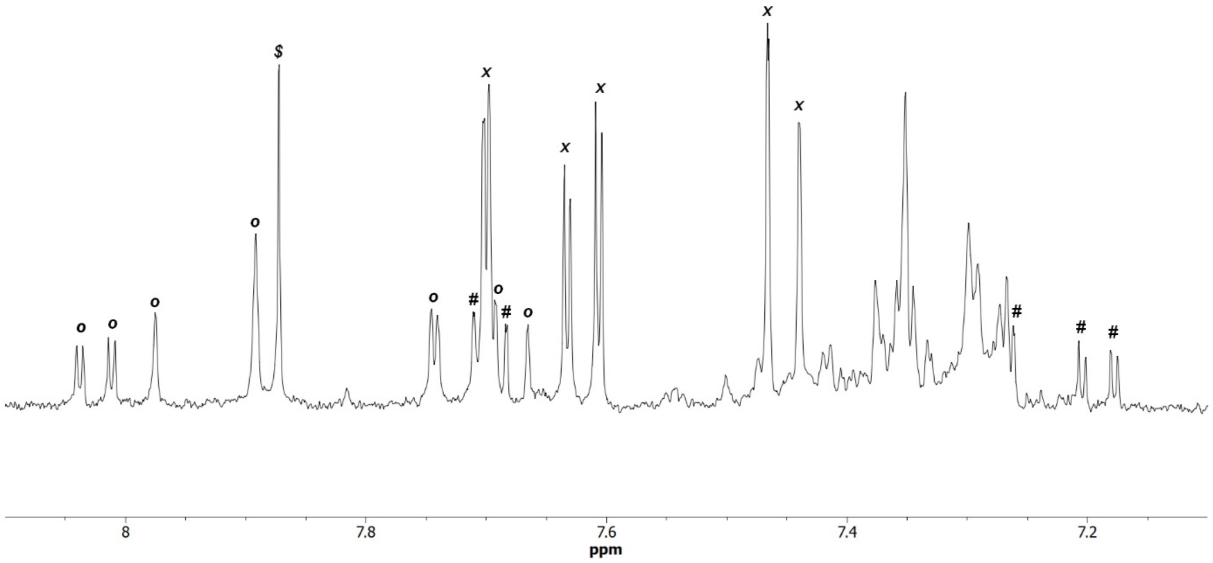

Figure S6. 7.1-8.1 ppm-range ^{1}H-NMR spectrum (300 MHz, NaOD/D_2O) of the digested **MOF 3**. For ^{1}H-NMR measurements, a MOF sample (≃ 10 mg) was suspended in D_2O (1 mL), and a solution of 40 wt% NaOD in D_2O (2 µL) was added. The solution was allowed to stand for 16 hours at room temperature, filtered on alumina and analyzed by NMR. Signals denoted "#" in the spectrum refer to BDC^{2-}-NH_2, "x" to BDC^{2-}-N_3, "*o*" to BDC^{2-}-TBTA, and signal denoted "$" refers to BDC^{2-}.

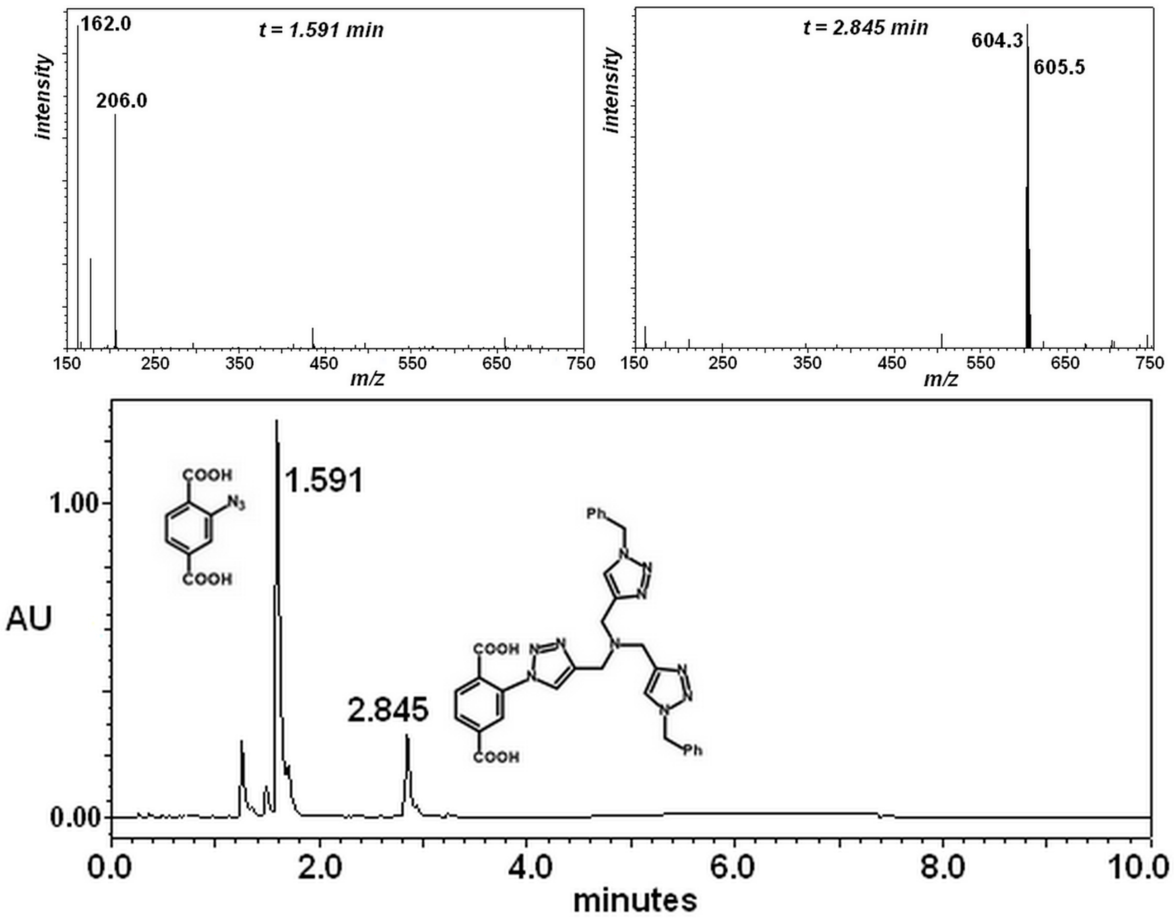

Figure S7. Mass spectra acquired in ESI negative mode of 2-azidoterephthalic acid (BDC-N_3, $C_8H_5N_3O_4$, MW 207.143) and of 2-[4-[[bis[(1-benzyltriazol-4-yl)methyl]amino]methyl] triazol-1-yl]terephthalic acid (BDC-TBTA, $C_{31}H_{28}N_{10}O_4$, MW 604.62) with the UPLC chromatogram (254 nm detection) of the digested products in ≃ 0.02 M NaOH of **MOF 3**; Retention times: 1.591 min for BDC-N_3 and 2.845 min for BDC-TBTA; Ratio: BDC-N_3 / BDC-TBTA 82/18.

Table S1[a]**.** "Recyclability" of **Cu@MOF1** and **Cu@MOF3** materials in the CuAAC reaction between benzyl azide and 3-phenyl-1-propyne at room temperature in methanol/water 95/5. Reaction conditions: 3-phenyl-1-propyne (0.86 mmol, 1 equiv), benzyl azide (1.03 mmol, 1.2 equiv), sodium ascorbate (10 mg, 6 mol%), MOF (10 mg), methanol/water 95/5 (1 mL). After each run of 24 h, the solid material was centrifuged, washed with ethyl acetate, dried under vacuum, and then used directly in the next run.

	**Cu@MOF1**		**Cu@MOF3**	
run number	conversion %	yield %	conversion %	yield %
#1	100	94	100	95
#2	100	96	100	93
#3	96	95	100	95
#4	97	93	95	91
#5	70	75	86	74
#6	34	31	52	46

[a]Histograms of these data are represented in **Fig.4**.

^{1}H- and ^{13}C-NMR data and spectra of compounds 4 – 7, 8 – 10

^{1}H-NMR spectra were recorded on a Bruker Avance 300 spectrometer at 300 MHz using $CDCl_3$ as solvent. The residual proton signal of the deuterated solvent was used as an internal reference ($CDCl_3$ δ = 7.26 ppm).

J-MOD ^{13}C- NMR spectra were recorded on a spectrometer on a Bruker Avance 300 spectrometer at 75 MHz. The carbon signal of the deuterated solvent was used as an internal reference ($CDCl_3$ δ = 77.0 ppm).

NMR data for **1,4-dibenzyl-1H-1,2,3-triazole** (**1**), **4-benzyl-1-dodecyl-1H-1,2,3-triazole** (**2**), **1-benzyl-4-phenyl-1H-1,2,3-triazole** (**3**) **and 1-benzyl-4-hexyl-1H-1,2,3-triazole** (**9**) are consistent with data already published [ref. S1-S2]

1-Dodecyl-4-phenyl-1H-1,2,3-triazole (**4**) [ref. S3]

Purified by flash chromatography: gradient petroleum ether/ethyl acetate 95/5 to 20/80 in 15 min. Yield: 94%

^{1}H NMR (300 MHz, $CDCl_3$) δ 7.83 (d, *J* = 7.3 Hz, 2H); 7.74 (s, 1H); 7.42 (t, *J* = 7.3 Hz, 2H); 7.32 (t, *J* = 7.3 Hz, 1H); 4.38 (t, *J* = 7.2 Hz, 2H); 1.93 (m, 2H); 1.29 (m, 18H); 0.87 (t, J = 7.0 Hz, 3H); ^{13}C NMR (75 MHz, $CDCl_3$) δ 147.7; 130.7; 128.8; 128.0; 125.6; 119.3; 50.4; 31.9; 30.3; 29.6; 29.5; 29.34; 29.28; 29.0; 26.5; 22.6; 14.1; LRMS (DCI-NH_3, M+H) Found: 314.2.

1-Benzyl-4-(3-methylphenyl)-1H-1,2,3-triazole (**5**)

Purified by flash chromatography: gradient petroleum ether/ethyl acetate 95/5 to 20/80 in 15 min. Yield: 98%

^{1}H NMR (300 MHz, $CDCl_3$) δ 7. 66 (br s, 2H); 7.56 (d, *J* = 7.5 Hz, 1H); 7.25-7.38 (m, 6H); 7.12 (d, *J* = 7.5 Hz, 1H); 5.54 (s, 2H); 2.37 (s, 3H); ^{13}C NMR (75 MHz, $CDCl_3$) δ 148.2; 138.4; 134.6; 130.3; 129.0; 128.8; 128.6; 128.6; 127.9; 126.3; 122.7; 119.5; 54.1; 21.3; HRMS (DCI-CH4, M+H) Calculated for $C_{16}H_{16}N_3$: 250.1344. Found: 340.1341.

9-(1-Benzyl-1H-1,2,3-triazol-4-yl)-9H-fluoren-9-ol (**6**)

Purified by flash chromatography: gradient petroleum ether/ethyl acetate 80/20 to 20/80 in 15 min. Yield: 93%

^{1}H NMR (300 MHz, $CDCl_3$) δ 7.62 (m, 2H); 7.58 (m, 2H); 7.36 (td, *J* = 7.5 Hz, 1.3 Hz, 2H); 7.24-7.33 (m, 5H); 7.17-7.20 (m, 2H); 7.14 (s, 1H); 5.39 (s, 2H); 3.83 (s, 1H); ^{13}C NMR (75 MHz, $CDCl_3$) δ 150.7; 147.8; 139.5; 134.3; 129.3; 128.9; 128.6; 128.2; 127.9; 124.8; 120.4; 120.1; 78.5; 54.0; HRMS (DCI-CH4, M+H) Calculated for $C_{22}H_{18}N_3O$: 340.1450. Found: 340.1445.

9-(1-Dodecyl-1H-1,2,3-triazol-4-yl)-9H-fluoren-9-ol (**7**)

Purified by flash chromatography: gradient petroleum ether/ethyl acetate 80/20 to 20/80 in 15 min. Yield: 97%

^{1}H NMR (300 MHz, $CDCl_3$) δ 7.62 (t, *J* = 8.3 Hz, 4H); 7.37 (td, *J* = 7.3 Hz, 1.2 Hz, 2H); 7.28 (td, *J* = 7.4 Hz, 1.1 Hz, 2H); 7.13 (s, 1H); 4.20 (t, *J* = 7.3 Hz, 2H); 3.88 (s, 1H); 1.79 (m, 2H); 1.25 (m, 18 H); 0.88 (t, *J* = 7.0 Hz, 3H); ^{13}C NMR (75 MHz, $CDCl_3$) δ 150.3; 148.0; 139.5; 129.3; 128.2; 124.8; 120.1; 78.5; 50.3; 31.8; 30.1; 29.5; 29.4; 29.3; 28.9; 26.4; 22.6; 14.1; HRMS (DCI-CH4, M+H) Calculated for $C_{27}H_{36}N_3O$: 418.2858. Found: 418.2668.

1-Benzyl-4-butyl-1H-1,2,3-triazole (**8**) [ref. 3]

Purified by flash chromatography: gradient petroleum ether/ethyl acetate 95/5 to 20/80 in 15 min. Yield: 98%

^{1}H NMR (300 MHz, $CDCl_3$) δ 7.22-7.38 (m, 5H); 7.18 (s, 1H); 5.47 (s, 2H); 2.67 (t, *J* = 7.5 Hz, 2H); 1.61 (m, 2H); 1.34 (m, 2H); 0.89 (t, *J* = 7.2 Hz, 3H); ^{13}C NMR (75 MHz, $CDCl_3$) δ 134.9; 128.9; 128.5; 127.8; 53.9; 31.4; 25.3; 22.2; 13.7; LRMS (DCI-NH_3, M+H) Found: 216.1.

1-Dodecyl-4-hexyl-1H-1,2,3-triazole (10)

Purified by flash chromatography: gradient petroleum ether/ethyl acetate 95/5 to 20/80 in 15 min. Yield: 96%

^{1}H NMR (300 MHz, $CDCl_3$) δ 4.26 (t, *J* = 7.2 Hz, 2H); 2.66 (t, *J* = 7.5 Hz, 2H); 1.84 (m, 2H); 1.62 (m, 2H); 1.21 (m, 27H); 0.84 (t, *J* = 6.8 Hz, 6H); ^{13}C NMR (75 MHz, $CDCl_3$) δ 148.3; 120.3; 50.1; 31.8; 31.5; 30.3; 29.5; 29.4; 29.3; 29.2; 28.9; 28.8; 26.4; 25.6; 22.6; 22.5; 14.0;13.9; HRMS (DCI-CH4, M+H) Calculated for $C_{20}H_{40}N_3$: 322.3222. Found: 322.3225.

References

S1. Menendez, C.; Chollet, A.; Rodriguez, F.; Inard, C.; Pasca, M.R.; Lherbet, C.; Baltas, M. Chemical synthesis and biological evaluation of triazole derivatives as inhibitors of InhA and antituberculosis agents. *Eur. J. Med. Chem.* **2012**, *52*, 275-283.

S2. Menendez, C.; Gau, S.; Lherbet, C.; Rodriguez, F.; Inard, C.; Baltas, M. Synthesis and biological activities of triazole derivatives as inhibitors of InhA and antituberculosis agents. *Eur. J. Med. Chem.* **2011**, 46, 5524-5531.

S3. Alonso, F.; Moglie, Y.; Radivoy, G.; Yus, M. Unsupported copper nanoparticles in the 1,3-dipolar cycloaddition of terminal alkynes and azides. *Eur. J. Org. Chem.* **2010**, 1875-1884.

Compound **4**

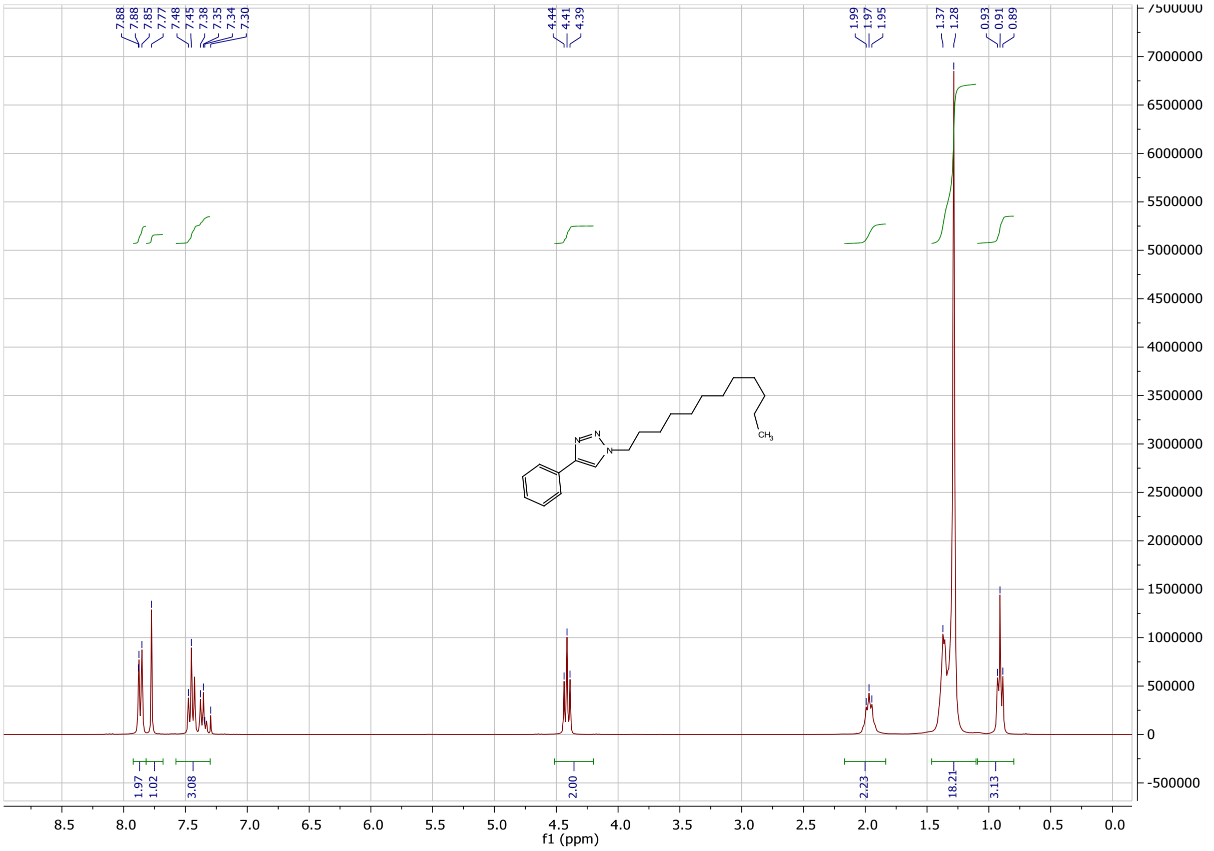

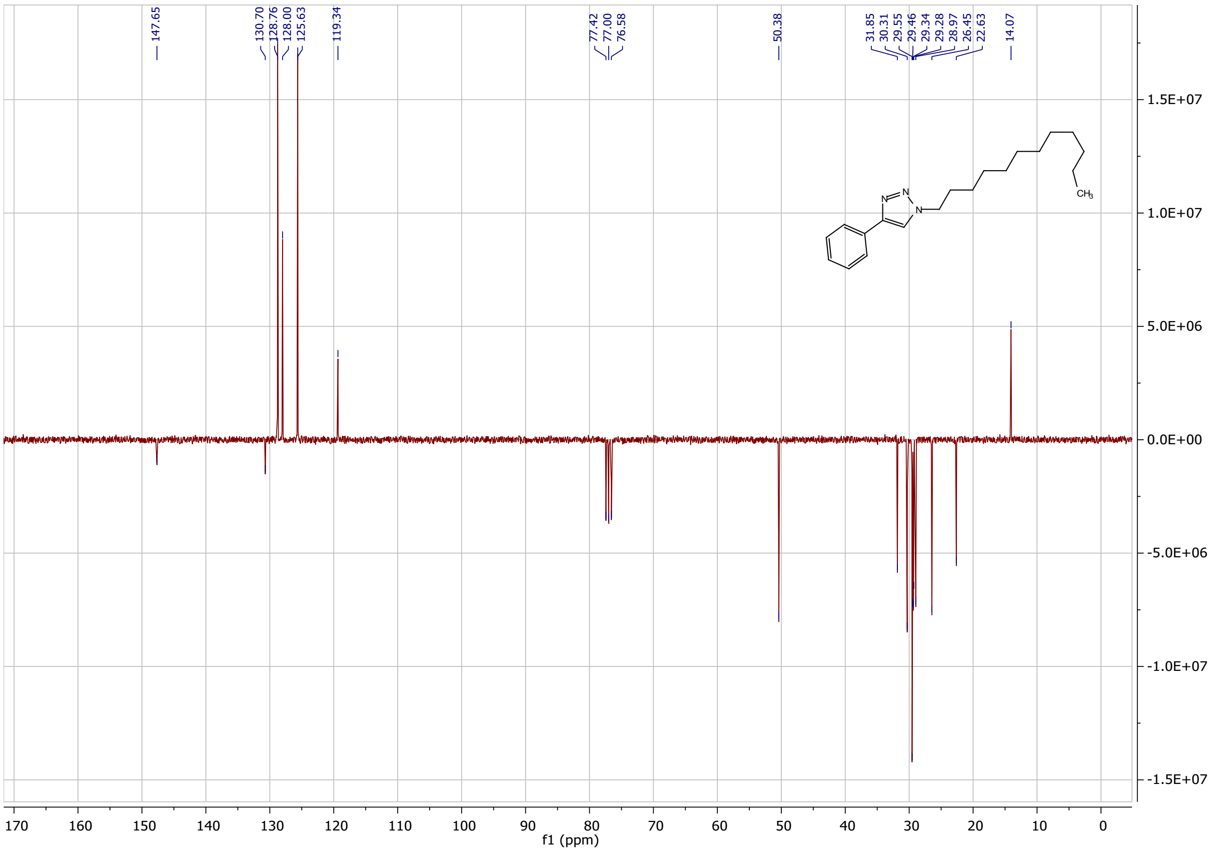

Compound **5**

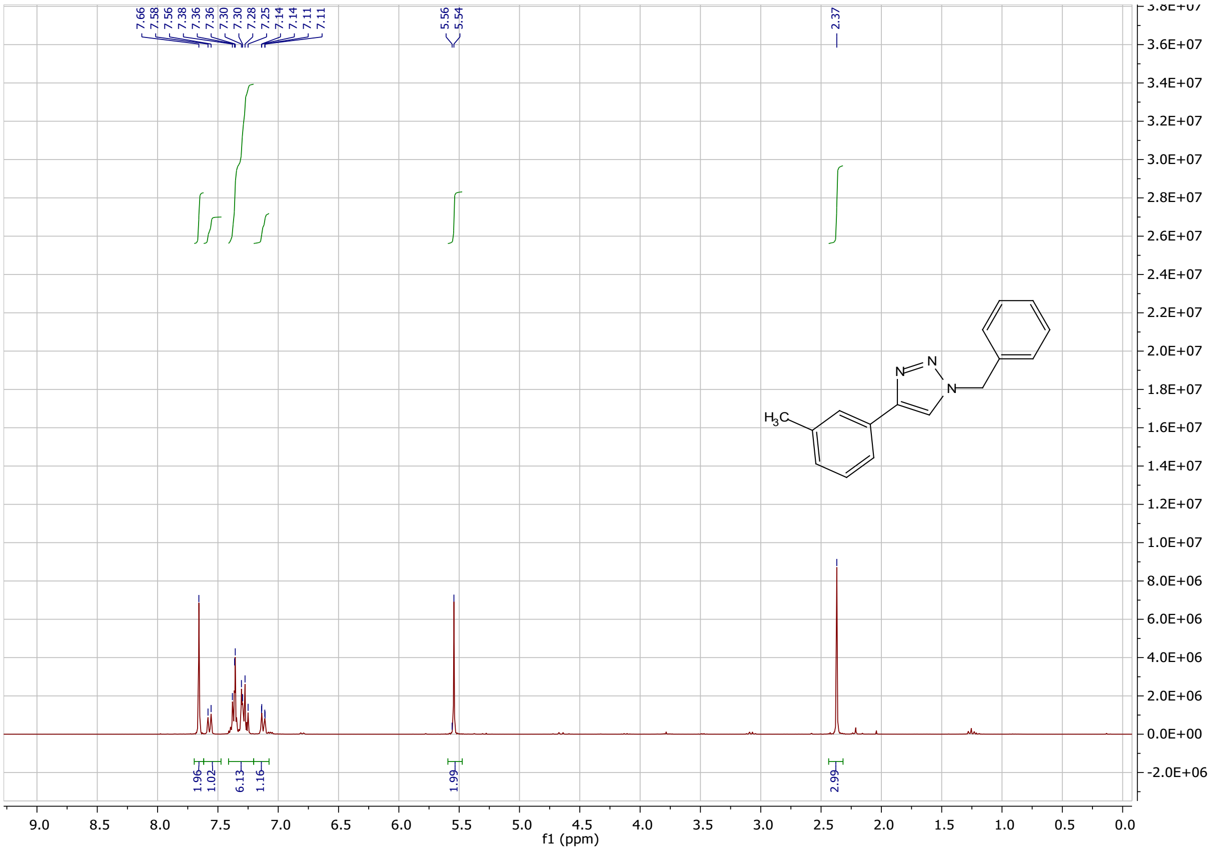

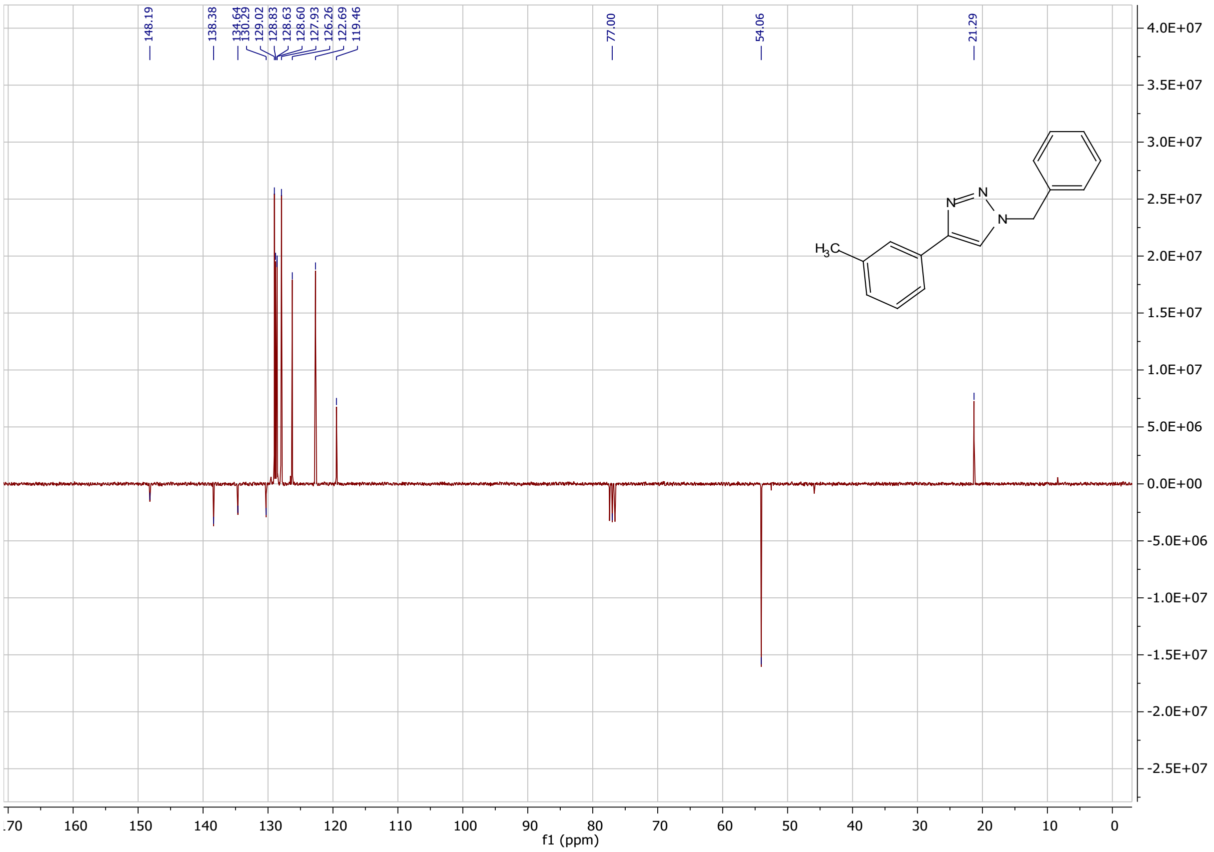

Compound **6**

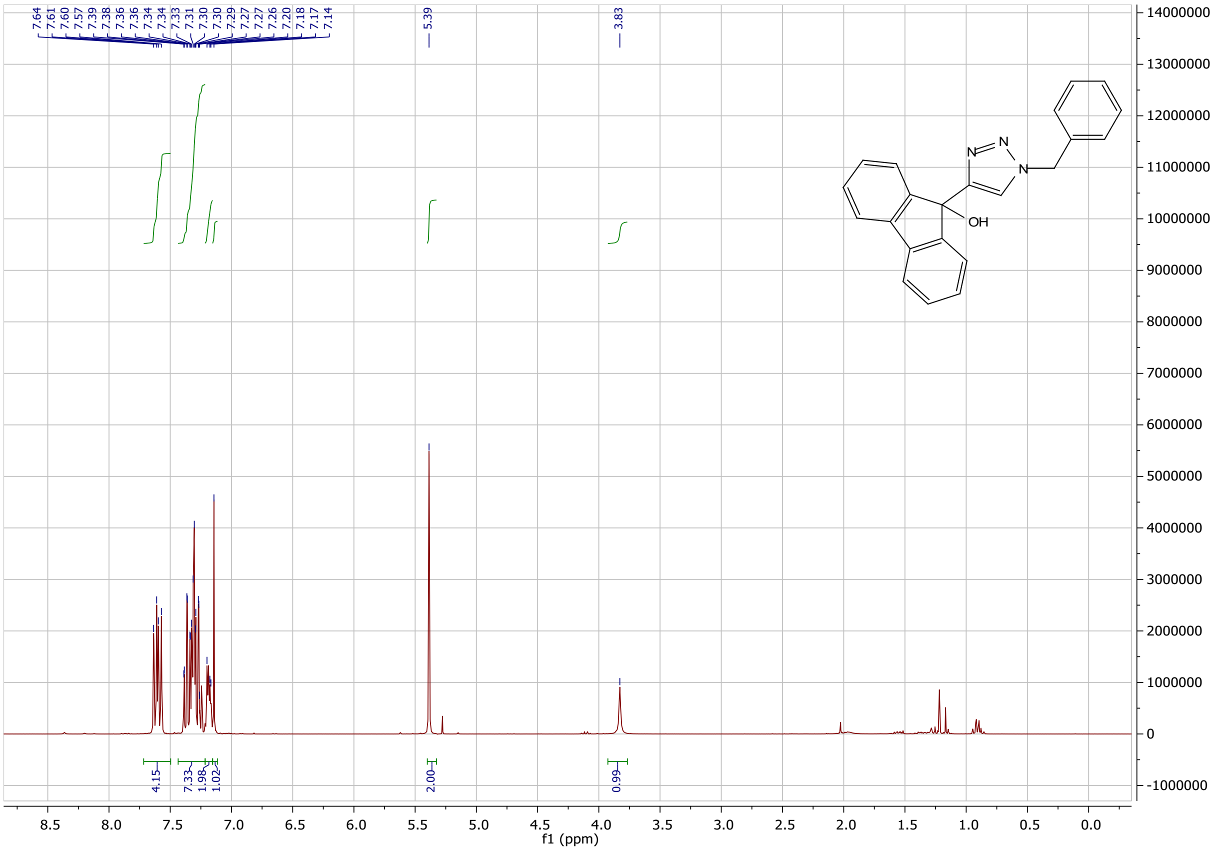
7.64
7.61
7.60
7.57
7.39
7.38
7.36
7.36
7.34
7.34
7.33
7.31
7.30
7.30
7.29
7.27
7.27
7.26
7.20
7.18
7.17
7.14
5.39
3.83
4.15
7.33
1.98
1.02
2.00
0.99
OH
N
N
N
f1 (ppm)

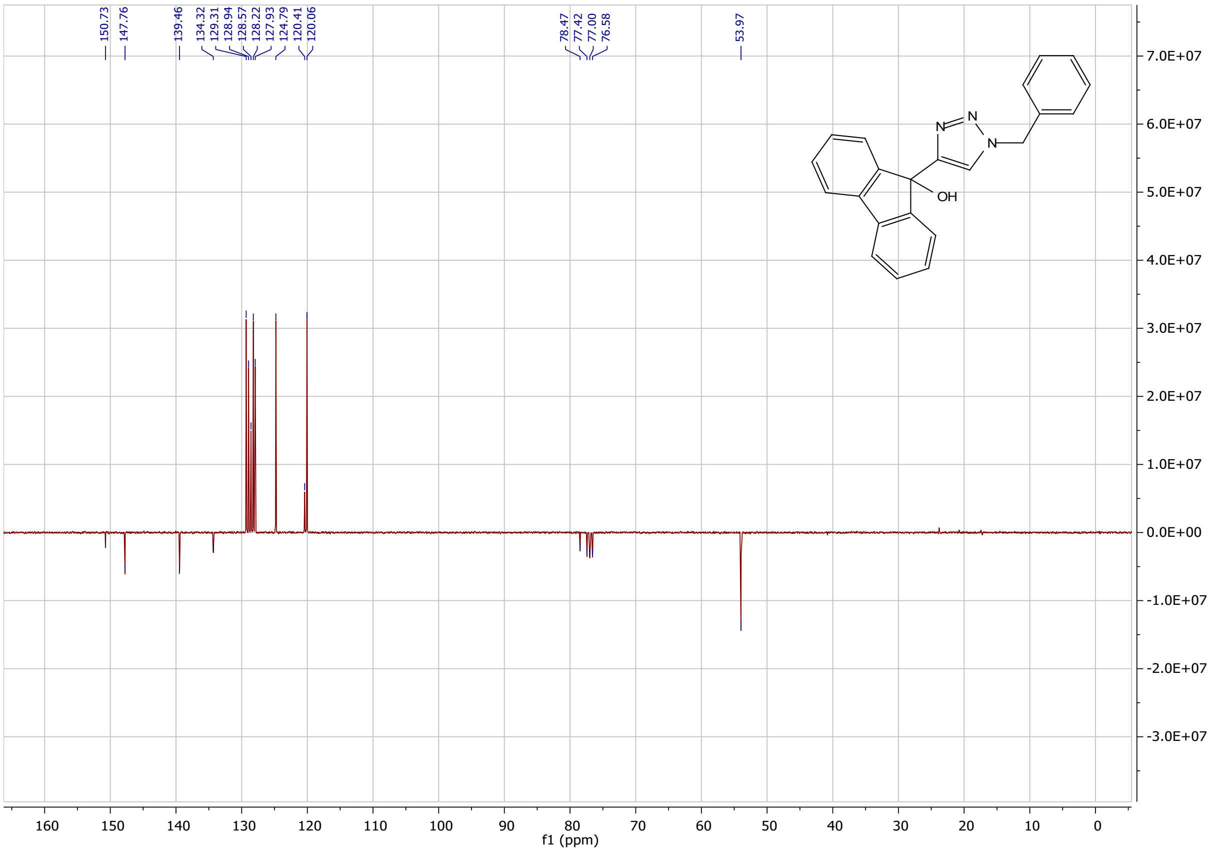
150.73
147.76
139.46
134.32
129.31
128.94
128.57
128.22
127.93
124.79
120.41
120.06
78.47
77.42
77.00
76.58
53.97
OH
N
N
N
f1 (ppm)

Compound **7**

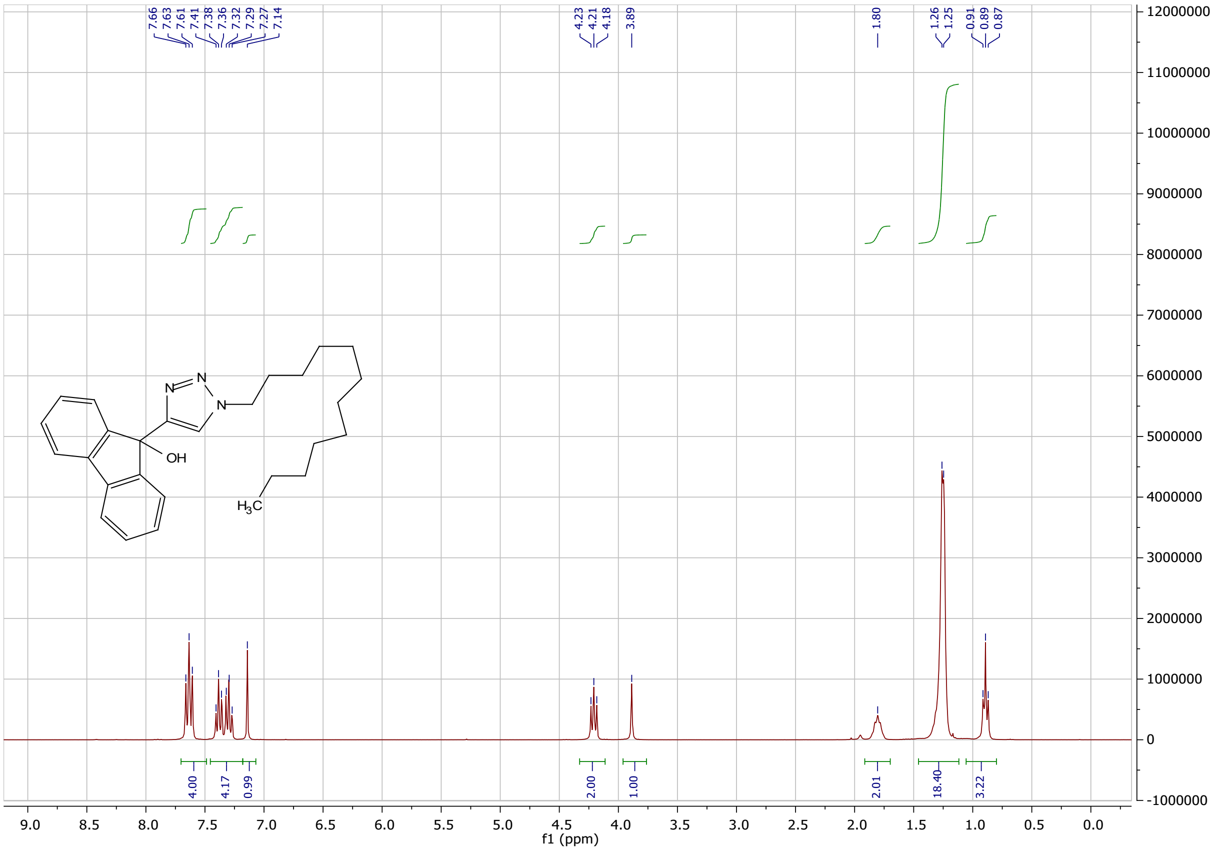
7.66
7.63
7.61
7.41
7.38
7.36
7.32
7.29
7.27
7.14
4.23
4.21
4.18
3.89
1.80
1.26
1.25
0.91
0.89
0.87
N
N
N
OH
H_3C
4.00
4.17
0.99
2.00
1.00
2.01
18.40
3.22
f1 (ppm)

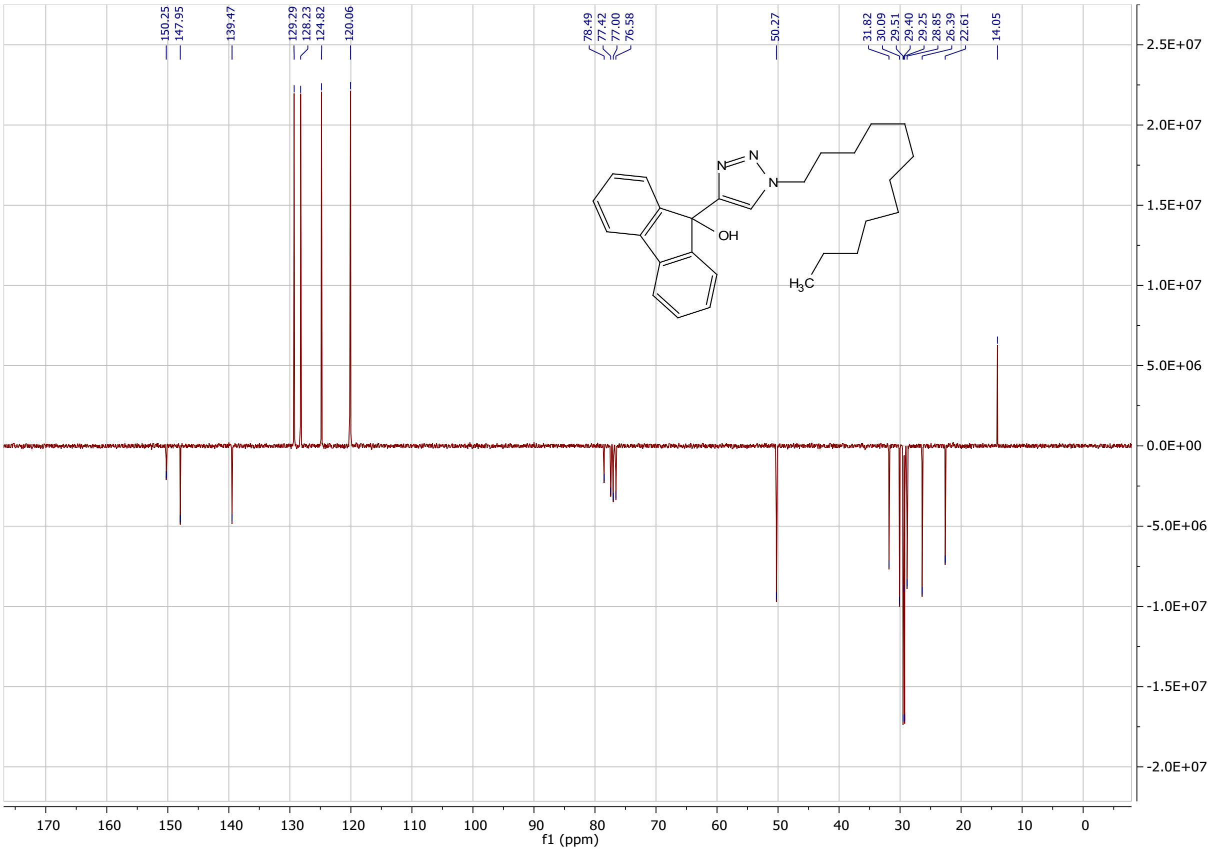
150.25
147.95
139.47
129.29
128.23
124.82
120.06
78.49
77.42
77.00
76.58
50.27
31.82
30.09
29.51
29.40
29.25
28.85
26.39
22.61
14.05
N
N
N
OH
H_3C
f1 (ppm)

Compound **8**

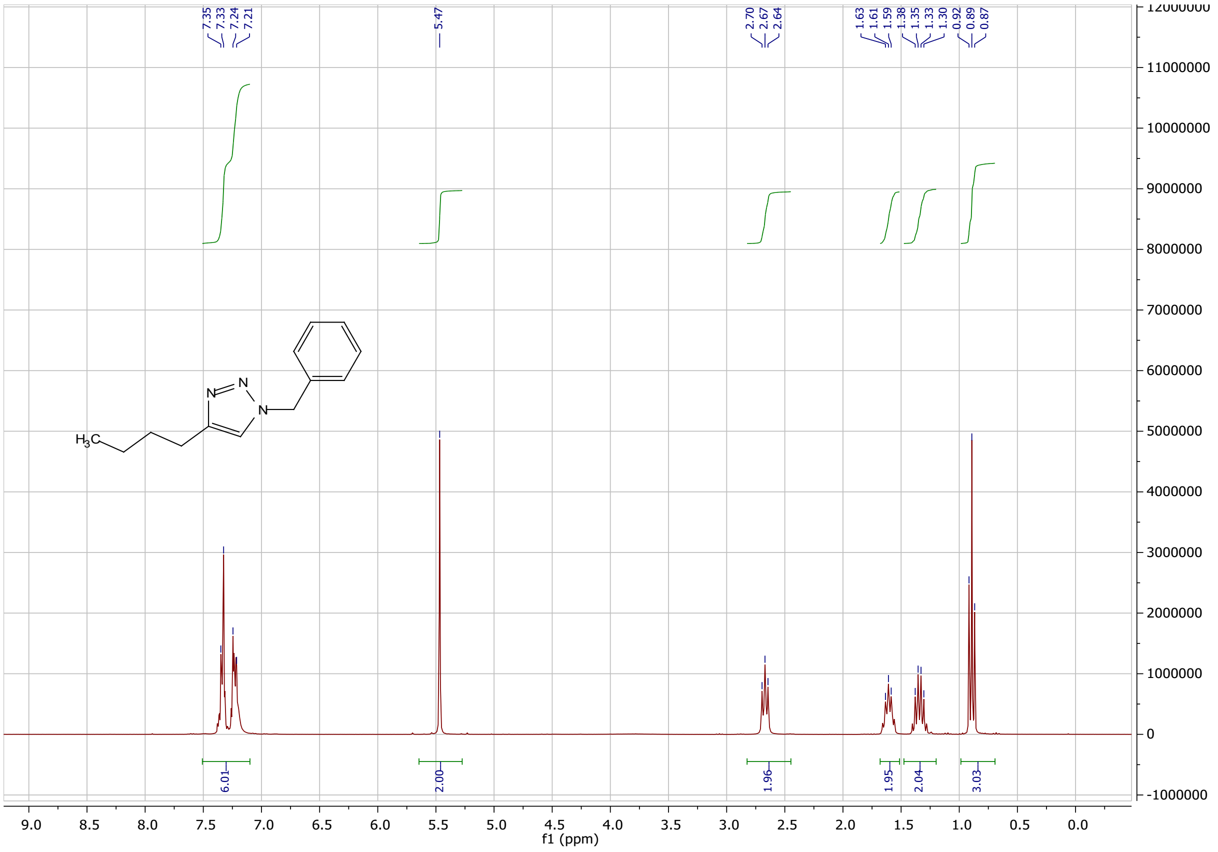

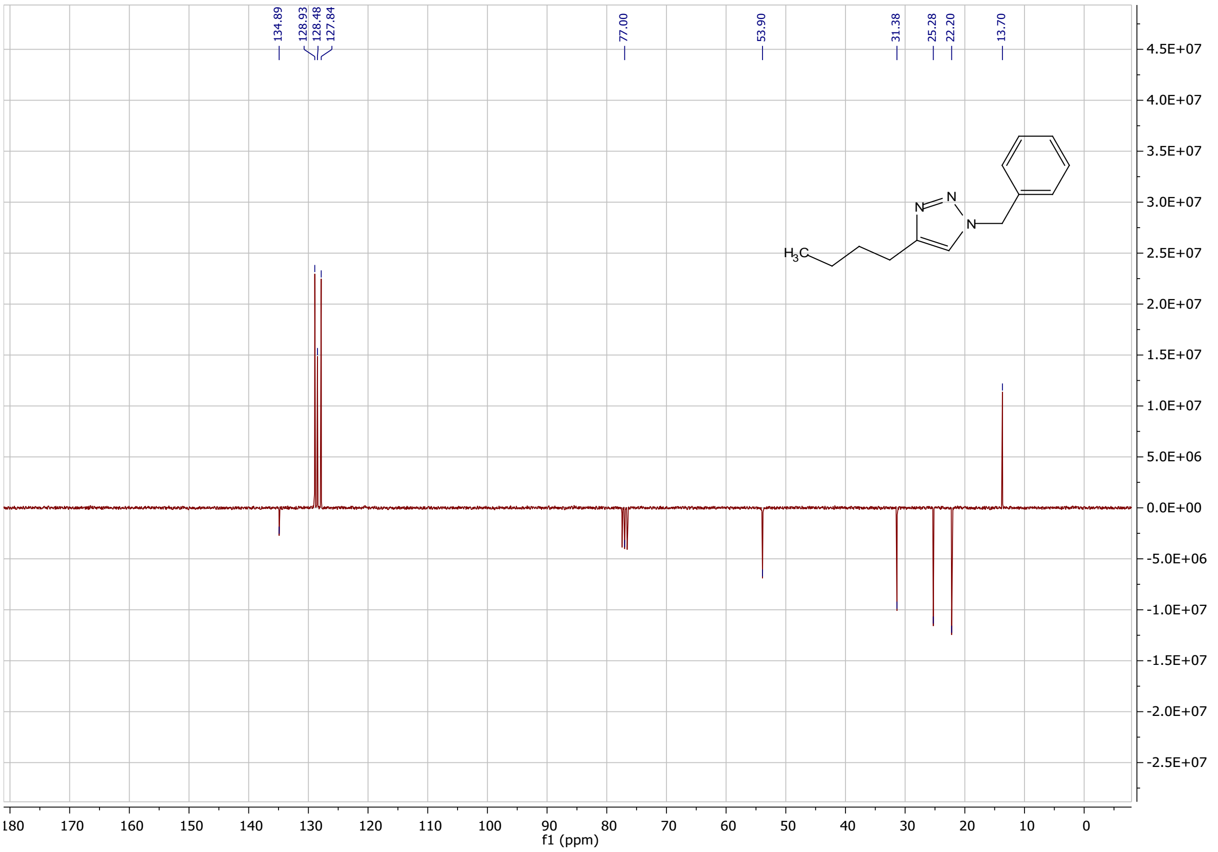

Compound **10**

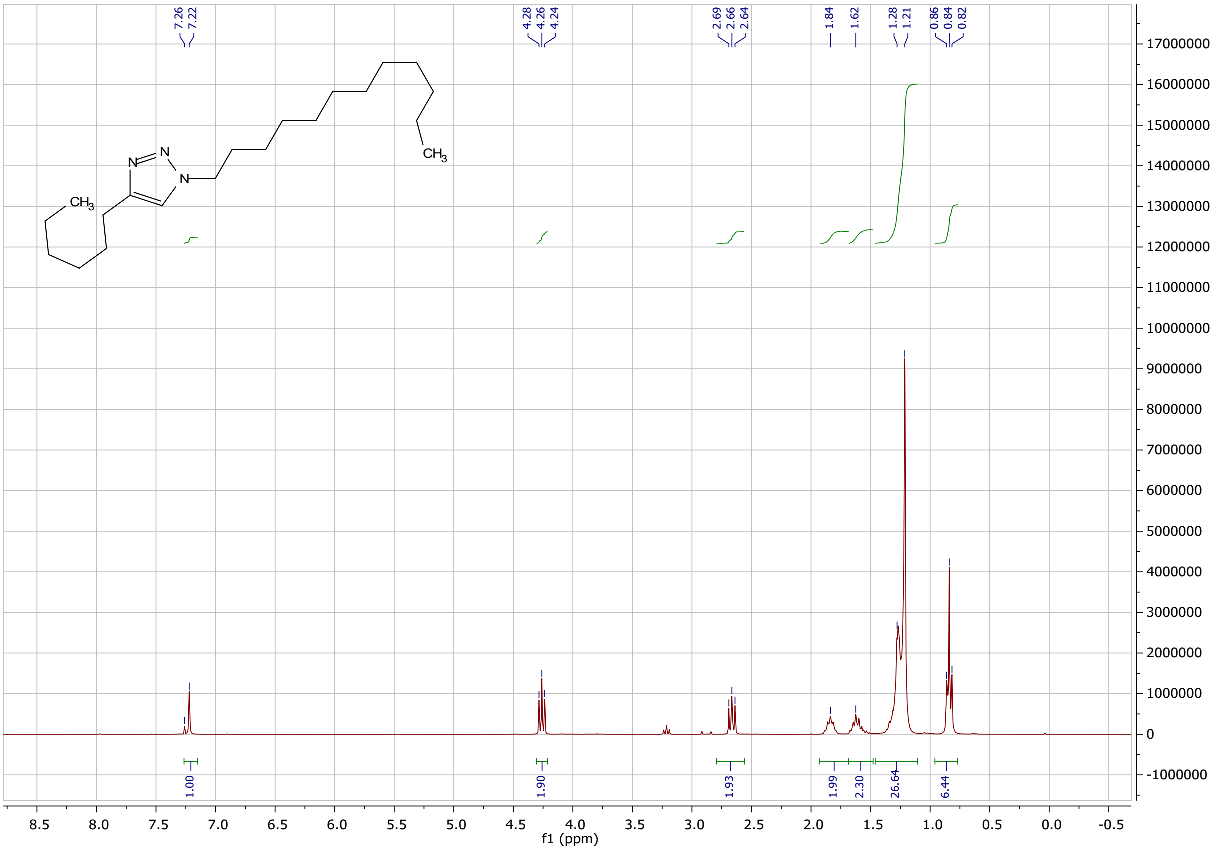
7.26
7.22
4.28
4.26
4.24
2.69
2.66
2.64
1.84
1.62
1.28
1.21
0.86
0.84
0.82
N
N
N
CH3
CH3
1.00
1.90
1.93
1.99
2.30
26.64
6.44
f1 (ppm)

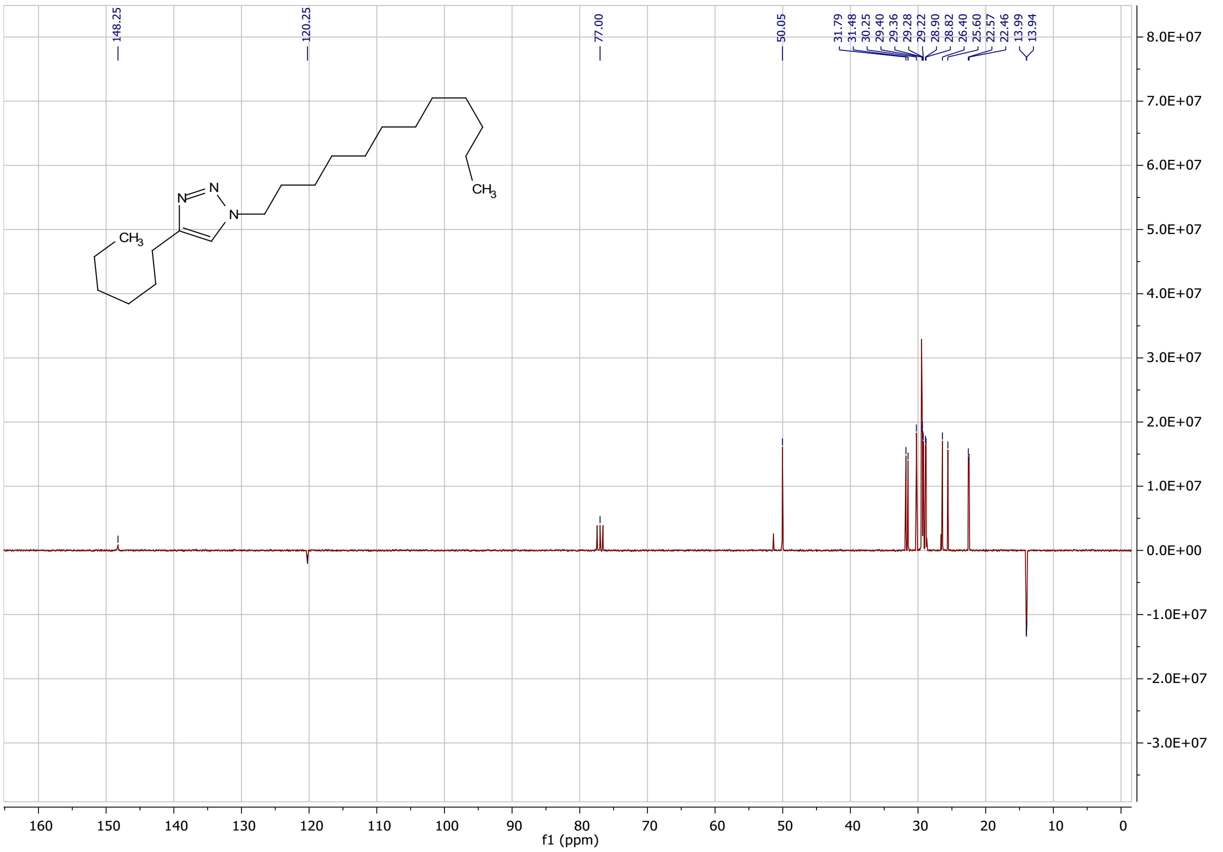
148.25
120.25
77.00
50.05
31.79
31.48
30.25
29.40
29.36
29.28
29.22
28.90
28.82
26.40
25.60
22.57
22.46
13.99
13.94
N
N
N
CH3
CH3
f1 (ppm)
